# Grain Nutrients Variability in Pigeonpea Genebank Collection and Its Potential for Promoting Nutritional Security in Dryland Ecologies

**DOI:** 10.3389/fpls.2022.934296

**Published:** 2022-07-11

**Authors:** Dhanapal Susmitha, Thiyagarajan Kalaimagal, Ramachandran Senthil, Mani Vetriventhan, Swaminathan Manonmani, Prabhakaran Jeyakumar, Bellie Anita, Surender Reddymalla, Pushpajeet L. Choudhari, Chetna A. Nimje, Ovais H. Peerzada, Venkata Narayana Arveti, Vania C. R. Azevedo, Kuldeep Singh

**Affiliations:** ^1^Genebank, International Crops Research Institute for the Semi-Arid Tropics (ICRISAT), Patancheru, India; ^2^Centre for Plant Breeding and Genetics, Tamil Nadu Agricultural University (TNAU), Coimbatore, India; ^3^Office of the Registrar, Tamil Nadu Agricultural University (TNAU), Coimbatore, India; ^4^Directorate of Open Distance Learning, Tamil Nadu Agricultural University (TNAU), Coimbatore, India; ^5^Charles Renard Analytical Laboratory, International Crops Research Institute for the Semi-Arid Tropics (ICRISAT), Patancheru, India; ^6^International Potato Center (CIP), Lima, Peru

**Keywords:** pigeonpea, protein, minerals, calcium, biofortification, landraces

## Abstract

Pigeonpea, a climate-resilient legume, is nutritionally rich and of great value in Asia, Africa, and Caribbean regions to alleviate malnutrition. Assessing the grain nutrient variability in genebank collections can identify potential sources for biofortification. This study aimed to assess the genetic variability for grain nutrients in a set of 600 pigeonpea germplasms conserved at the RS Paroda Genebank, ICRISAT, India. The field trials conducted during the 2019 and 2020 rainy seasons in augmented design with four checks revealed significant differences among genotypes for all the agronomic traits and grain nutrients studied. The germplasm had a wider variation for agronomic traits like days to 50% flowering (67–166 days), days to maturity (112–213 days), 100-seed weight (1.69–22.17 g), and grain yield per plant (16.54–57.93 g). A good variability was observed for grain nutrients, namely, protein (23.35–29.50%), P (0.36–0.50%), K (1.43–1.63%), Ca (1,042.36–2,099.76 mg/kg), Mg (1,311.01–1,865.65 mg/kg), Fe (29.23–40.98 mg/kg), Zn (24.14–35.68 mg/kg), Mn (8.56–14.01 mg/kg), and Cu (7.72–14.20 mg/kg). The germplasm from the Asian region varied widely for grain nutrients, and the ones from African region had high nutrient density. The significant genotype × environment interaction for most of the grain nutrients (except for P, K, and Ca) indicated the sensitivity of nutrient accumulation to the environment. Days to 50% flowering and days to maturity had significant negative correlation with most of the grain nutrients, while grain yield per plant had significant positive correlation with protein and magnesium, which can benefit simultaneous improvement of agronomic traits with grain nutrients. Clustering of germplasms based on Ward.D2 clustering algorithm revealed the co-clustering of germplasm from different regions. The identified top 10 nutrient-specific and 15 multi-nutrient dense landraces can serve as promising sources for the development of biofortified lines in a superior agronomic background with a broad genetic base to fit the drylands. Furthermore, the large phenotypic data generated in this study can serve as a raw material for conducting SNP/haplotype-based GWAS to identify genetic variants that can accelerate genetic gains in grain nutrient improvement.

## Introduction

Malnutrition exists in most countries and across all socioeconomic classes. Undernutrition, micronutrient deficiency, and obesity are the implications of a nutritiously imbalanced diet (Food and Agriculture Organization [FAO], 2014). A healthy diet should meet the recommended dietary allowance of 54 g (men) and 46 g (women) protein, 1,000-mg phosphorus, 2,000-mg potassium, 1,000-mg calcium, 2-mg copper, 440 mg (men), and 370-mg (women) magnesium, 4-mg manganese, 19-mg (men) and 29-mg (women) iron, and 17-mg (men) and 13-mg (women) zinc per day (ICMR-NIN, 2020). Severe protein deficiency characterized by Kwashiorkor is widespread in developing countries. Similarly, micronutrient deficiencies of common occurrence are iron, vitamin A, and iodine (World Health Organization [WHO], 2021). Anemia outbreaks as a result of iron deficiency, and, globally, 1.8 billion people were anemic as of 2019, with South Asia, West Sub-Saharan Africa, and Central Sub-Saharan Africa regions having high prevalence (Safiri et al., 2021). Furthermore, poverty and malnutrition are interrelated to each other. Poverty compromises the dietary quality of food and results in the intake of inexpensive starchy food (Siddiqui et al., 2020). Imbalanced energy and protein intake result in protein-energy malnutrition (Bailey and West, 2015). The dietary protein intake can be of plant or animal origin. Furthermore, the source of protein origin has an impact on human health. Substituting foods rich in animal protein with plant protein can prolong longevity (Song et al., 2017; Naghshi et al., 2020). Animal protein production disturbs environmental sustainability (Aiking and De Boer, 2020), and its consumption also adds to the spread of zoonotic diseases (Andreoli et al., 2021). Comparatively, plant protein exerts less pressure on the environment. The only limitation associated with plant protein is the poor protein quality that is affected by the anti-nutritional factors contained in it, which in turn reduces the bio-availability of minerals (Pele et al., 2016; Ahnen et al., 2019).

Grain legumes were identified as the cheapest source of good quality protein (Dahiya et al., 1977). The nutritional profile states that legumes have two times the quantity of cereal protein, with no cholesterol and less fat (other than soybean and groundnut), and serves as a rich source of essential minerals, namely, Zn, Fe, Ca, Se, P, Cu, K, Mg, and Cr. The consumption of grain legumes dates to 5500 BC and is the second most consumed food crop across the globe next to cereals (Kouris-Blazos and Belski, 2016). Other than serving as high-quality food and feed, grain legumes defend the globe with reduced emission of greenhouse gases (5–7 times lesser than other crops). Carbon sequestration and atmospheric nitrogen fixation by grain legumes help to diversify crop cultivation, and reduced external inputs usage finds itself as a potential crop for sustainable agriculture (Stagnari et al., 2017).

Pigeonpea, also called as red gram, is a climate-resilient drylands legume and is widely cultivated in semiarid regions (Mula and Saxena, 2010). Globally, ∼5. million tonnes were produced from a planted area of 6.1 million hectares. Five countries, namely, India (82%), Myanmar (7%), Malawi (4%), Kenya (2%), and Tanzania (2%) account for 97% of the total cultivated area (FAOSTAT, 2021). Pigeonpea serves a variety of purposes, such as food, forage, feed, and meal for animals, piggery and fishery, fuel wood, green manure, barrier crop, rearing of lac insects, and roof thatches (Harinder et al., 1999; Upadhyaya et al., 2007; Mula and Saxena, 2010; Ohizua et al., 2017; Wangari et al., 2020). Nutritionally, pigeonpea grain is rich in protein, Ca, Mn, and crude fiber (Saxena et al., 2010). The variability for protein content, reported in previous studies, varied from 16.7 to 26.8% (Amarteifio et al., 2002; Sekhon et al., 2017; Obala et al., 2018; Cheboi et al., 2019; Choi et al., 2020; Jawalekar et al., 2020), whereas, in wild species, the range is from 16.3 to 33.8% (Upadhyaya et al., 2013). Very few studies enumerated mineral composition in pigeonpea (Singh et al., 1984; Oshodi et al., 1993; Amarteifio et al., 2002; Mishra and Acharya, 2018). Dahiya et al. (1977) reported the substantial influence of environment on protein, partial dominance of low protein over high protein, and negative correlation between seed yield and protein content. Furthermore, the nutrient accumulation varied with the seed developmental stage, Fe and Zn are rich at the green stage, whereas protein, starch, Mn, and Ca are high at the grain stage (Singh et al., 1984). Unlike cereals, the Fe and Zn are enriched in the cotyledons; thus the processing does not affect the availability of these minerals (Susmitha et al., 2022). Nutrient improvement in pigeonpea was done utilizing few wild species. Reddy et al. (1979) utilized wild species *Atylosia* from the tertiary gene pool to develop high protein lines, while Sharma et al. (2020) utilized *Cajanus platycarpus* to broaden the variability available for agronomic and grain nutritional traits. Saxena et al. (1987) identified high protein lines (HPL 2, HPL 7, HPL 40, and HPL 51) with 27–29% protein from five intergeneric crosses and mentioned the variable association between seed size and protein across crosses. Compared to normal lines (C 11 and ICPL 211), a hike of nearly 20% protein was observed in high protein lines (HPL 8 and HPL 40; Singh et al., 1990).

At ICRISAT, research on pigeonpea is focused primarily on the development of mid-early, early, and super-early varieties/hybrids with high yielding potential to attain self-sufficiency in the target areas. However, the identification of genetic resources with superior grain nutrients can support pigeonpea biofortification and add nutritional security. The ICRISAT genebank conserves 13,787 pigeonpea germplasm.^1^ This study was planned to characterize 600 diverse pigeonpea accessions for grain nutrients and important agronomic traits in 2 cropping years, with the objectives (i) to assess the variability for agronomic and grain nutritional traits, (ii) to understand the association between and among the agronomic traits and grain nutrients, and (iii) to identify trait-specific and multi-nutrient dense germplasm.

## Materials and Methods

### Genetic Resources

**FIGURE 1:**
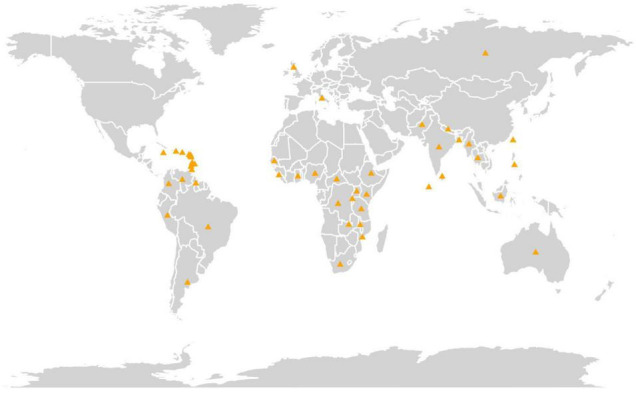
A map depicting the country of collection of the 600 pigeonpea accessions and four checks conserved at Genebank, ICRISAT, India.

The experimental material comprised 600 pigeonpea accessions conserved at Rajendra Singh Paroda Genebank, ICRISAT, India, along with four checks (Supplementary Table 1). The complete passport data of the germplasm are available in the ICRISAT Genebank database (see text footnote 1). These accessions represent 48 countries (Figure 1) across the globe, and five geographical regions, namely, Asia (357), Africa (148), America (80), Europe (11), and Oceania (4). The map depicting the country of collection was generated using R statistical software v.4.0.2 (R Core Team, 2021) using the “map” (South, 2011) and “ggplot2” (Wickham, 2016) packages. Out of 600 pigeonpea accessions, 577 were landraces, 19 improved cultivars/breeding lines, and four wild species accessions, *Cajanus acutifolius* (2), *C. cajanifolius* (1), and *C. sericeus* (1). ICP 11543 (an extra-early cultivar known as Pragati), ICP 6971 (an early cultivar known as UPAS-120), ICP 8863 (a medium duration cultivar, popularly known as Maruti), and ICP 7221 (a well-known long-duration cultivar named as Gwalior 3) were used as checks. Among these, ICP 11543 was developed by pedigree selection from the T 21 × JA 277 cross, while the remaining were developed through selection from the germplasm.

### Field Experimental Design and Soil Properties

The experiment was laid in an augmented design with 20 blocks. Each block comprised of 30 test entries and four checks. Sowing was done in the last week of July in 2 cropping years, i.e., 2019 and 2020 at ICRISAT Patancheru, India (located at 17.51^°^N latitude, 78.27^°^E longitude, and 545 m above the mean sea level) in alfisols. Each accession was sown in a 4-meter row with an inter-row spacing of 75 cm and plant-plant spacing of 20–25 cm. As per the USDA soil taxonomy, the soil belongs to the fine loamy-mixed isohyperthermic family of Udic Rhodustalf. The first 30-cm soil of the experimental field in the 2019 rainy season had 7.22 pH, 0.07 dS/m EC, 0.42% organic matter, 7.5 mg/kg P, 67 mg/kg K, 1,116 mg/kg exchangeable Ca, 368 mg/kg exchangeable Mg, 6.1 mg/kg Fe, 1.39 mg/kg Zn, 1.34 mg/kg Cu, and 18.53 mg/kg Mn, and the 2020 rainy season had 6.97 pH, 0.08 dS/m EC, 0.45% organic matter, 18.67 mg/kg P, 79 mg/kg K, 1,057 mg/kg exchangeable Ca, 340 mg/kg of exchangeable Mg, 8.93 mg/kg Fe, 4.24 mg/kg Zn, 1.25 mg/kg Cu, and 17.54 mg/kg Mn.

### Agronomic Practices and Phenotyping

The agronomic practices started with the basal application of DAP (diammonium phosphate) at a rate of 100 kg/hectare. Thinning was practiced 21 days after sowing to maintain optimum plant density. Optimum field conditions were maintained following standard package of practises. Agronomic traits recorded were days to 50% flowering, days to maturity, 100-seed weight, and grain yield per plant. Days to 50% flowering was recorded on a plot basis. Days to maturity and grain yield per plant were recorded on a single-plant basis (5–21 plants) and averaged to represent the accession. The 100-seed weight was recorded from a random sample of 100 seeds drawn from the bulked single-plant yield of each accession. Grain nutrients analyses were performed on 598 accessions, while two checks (ICP 11543 and ICP 6971) and four accessions having poor germination/plant stand were excluded.

### Grain Nutrients Estimation

The grain nutrients estimated in the study were protein, P, K, Ca, Cu, Mg, Mn, Fe, and Zn. Clean and dust-free grain samples weighing 15 g were taken from the bulked single-plant yield of each accession in each cropping year for grain nutritional analysis. The grain samples were submitted following the augmented design. The grain nutrients estimation was done at Charles Renard Analytical laboratory, ICRISAT, India. Protein estimation was done by digesting the grain sample by the sulfuric acid-selenium digestion method and analyzing the digests in a continuous flow autoanalyzer to obtain the total N value from which protein (%) is calculated by multiplying the total N with a 6.25 conversion factor (Sahrawat et al., 2002). Estimation of P, K, Ca, Cu, Mg, Mn, Fe, and Zn was done by digesting the plant samples with the nitric acid – hydrogen peroxide digestion method and analyzing the digests in Microwave Plasma Atomic Emission Spectrometry (MP-AES; Wheal et al., 2011).

### Statistical Analysis

The components of variances for four agronomic and nine-grain nutritional traits for the individual years and pooled data over 2 years were analyzed by adopting the linear mixed model in residual maximum likelihood (REML) in GenStat 19 (VSN International, 2019). For the individual years, entry and block were assigned as random effects, whereas, in pooled data over years, the cropping year was kept fixed, and the factors, namely, entry, cropping year, and block were assigned random. Variance due to genotype ($\sigma_{g}^{2})$, genotype × environment ($\sigma_{g\times e}^{2})$, and error ($\sigma_{e}^{2})$ was estimated, while the significance of cropping years was tested by Wald’s statistics (Wald, 1943). Heritability in broad sense for individual and pooled data over cropping years for each trait was estimated and categorized based on Johnson et al. (1955). Best linear unbiased predictors (BLUPs; Schönfeld and Werner, 1986) obtained for all the traits for each accession in each cropping year, and pooled analyses over cropping years were used for all downstream analyses. The accessions were broadly classified into three maturity groups as early (≤150 days to maturity), medium (151–180 days to maturity), and late (>180 days to maturity; Reddy, 1990). Newman–Keuls test (Newman, 1939; Keuls, 1952) and Levene’s test (Levene, 1960) were used to compare the mean and test the homogeneity of variances in different groups formed based on the geographical region and maturity using R packages “agricolae” (de Mendiburu, 2021) and “car” (Fox and Weisberg, 2019). Histogram and a density graph depicting the distribution of agronomic and grain nutrients in each cropping year, geographical region, and maturity group were visualized using the package “ggplot2” (Wickham, 2016). The correlation coefficients among the agronomic and grain nutritional traits were performed using the native R function “cor ()” and visualized using the “corrplot” (Wei and Simko, 2021) package. The phenotypic distance matrix for four agronomic traits and nine-grain nutrients was constructed following the Gower’s dissimilarity method using the R package “vegan” (Oksanen et al., 2020) and the dendrogram constructed based on the Ward.D2 method (Murtagh, 2014) using the R package “cluster” (Maechler et al., 2021), with a heatmap depicting the agronomic performance and grain nutrients content of each accession of the cluster using the package “heatmap3” (Zhao et al., 2021). The cluster means were compared using the Newman–Keuls test (Newman, 1939; Keuls, 1952). The circular stacked barplot depicting the contribution of each region to the sub-cluster was constructed using the “ggplot2” (Wickham, 2016) package. The nutrient-specific and multi-nutrient dense accessions were identified based on *per se* performance and superiority to the best check.

## Results

### Components of Variance

The REML ANOVA indicated that the variance due to genotypes was highly significant (*p* ≤ 0.01) for all agronomic and grain nutrients for individual cropping years and pooled analysis over cropping years except for P, K, and Mn of the 2019 rainy season (Table 1). The variance due to Genotype × Cropping year interaction ($\sigma_{g\times e}^{2})$ was significant for days to 50% flowering, 100-seed weight, grain yield per plant, and for grain nutrients, namely, protein, Fe, Zn, Mg, Cu, and Mn (*p* ≤ 0.05) while insignificant for days to maturity, P, K, and Ca. However, the variance due to genotype ($\sigma_{g}^{2})$ was higher than the G × E variance ($\sigma_{g\times e}^{2})$ for days to 50% flowering, 100-seed weight, Mg, Cu, Mn, and Zn, but it was reverse for grain yield per plant, protein, and Fe. Wald’s statistics for the environment (cropping years) revealed a significant difference between the cropping years ($\sigma_{e}^{2}$) for all agronomic and grain nutritional traits except for Ca.

**TABLE 1:** Variance, mean, range, co-efficient of variation (CV %), least significant difference (LSD_0.05_), and heritability (broad sense) for agronomic and grain nutritional traits of pigeonpea accessions evaluated during 2019 and 2020 rainy seasons at ICRISAT, Hyderabad, India.

Trait	Environment ($\sigma_{e}^{2})$		Genotype ($\sigma_{g}^{2})$	G × E ($\sigma_{g\times e}^{2})$	Mean ± SD	Range	CV (%)	LSD_0.05_	*h* ^2^
Days to 50% flowering	Pooled	1730.62**	245.12**	17.87**	124 ± 15.6	67–166	3.84	13.21	0.92
	2019		284.60**		131 ± 16.5^a^	71–171	4.63	16.89	0.89
	2020		219.34**		116 ± 14.8^b^	62–160	1.38	4.46	0.99
Days to maturity	Pooled	2295.27**	230.11**	8.09	174 ± 14.9	112–213	2.86	13.84	0.93
	2019		259.97**		182 ± 15.4^a^	120–221	3.10	15.68	0.89
	2020		197.68**		166 ± 14.5^b^	105–206	1.31	6.03	0.98
100-seed weight (g)	Pooled	408.14**	7.26**	0.69**	10.10 ± 2.7	1.69–22.17	5.17	1.45	0.94
	2019		6.65**		10.67 ± 3.0^a^	1.96–23.28	10.66	3.16	0.84
	2020		6.32**		9.52 ± 2.6^b^	1.41–21.05	6.08	1.61	0.95
Grain yield per plant(g)	Pooled	28.22**	47.65**	51.67**	32.37 ± 6.9	16.54–57.93	22.22	19.98	0.48
	2019		112.34**		30.63 ± 7.6^b^	13.13–61.45	18.63	15.83	0.78
	2020		106.90**		34.10 ± 7.7^a^	13.73–61.81	19.28	18.31	0.71
Protein (%)	Pooled	21.87**	1.10**	1.47*	26.99 ± 1.0	23.35–29.50	6.90	5.17	0.31
	2019		2.67**		27.30 ± 1.2^a^	23.07–30.60	6.29	4.77	0.48
	2020		1.57**		26.69 ± 1.0^b^	23.54–29.11	5.21	3.86	0.45
Phosphorus (%)	Pooled	140.59**	0.0006**	0.0008	0.43 ± 0.02	0.36–0.50	12.91	0.15	0.25
	2019		0.0013		0.45 ± 0.02^a^	0.35–0.54	12.09	0.15	0.31
	2020		0.0006**		0.41 ± 0.02^b^	0.35–0.48	4.71	0.05	0.61
Potassium (%)	Pooled	408.10**	0.0016**	0.0002	1.50 ± 0.02	1.43–1.63	5.34	0.22	0.33
	2019		0.0019		1.46 ± 0.02^b^	1.39–1.58	5.00	0.20	0.26
	2020		0.0029**		1.55 ± 0.02^a^	1.48–1.67	3.78	0.16	0.46
Calcium (mg/kg)	Pooled	1.00	38700**	3357	1542.80 ± 171.2	1042.36–2099.76	11.13	476.83	0.70
	2019		42988**		1537.58 ± 173.1^a^	1025.98–2110.34	10.87	463.95	0.61
	2020		41289**		1548.03 ± 170.3^a^	1058.74–2089.18	9.53	410.15	0.65
Magnesium (mg/kg)	Pooled	37.93**	7314**	2287*	1530.20 ± 78.2	1311.01–1865.65	5.14	218.42	0.63
	2019		13443**		1513.73 ± 82.5^b^	1283.83–1835.19	3.62	152.18	0.82
	2020		7932**		1546.66 ± 77.6^a^	1338.19–1896.11	4.57	196.48	0.61
Copper (mg/kg)	Pooled	371.6**	0.75**	0.30**	11.17 ± 0.8	7.72–14.20	6.74	2.09	0.63
	2019		0.85**		10.65 ± 0.9^b^	6.58–13.45	8.58	2.54	0.51
	2020		0.84**		11.69 ± 0.8^a^	8.86–15.05	5.30	1.72	0.69
Manganese (mg/kg)	Pooled	139.73**	0.41**	0.38**	10.40 ± 0.6	8.56–14.01	7.55	2.18	0.45
	2019		0.29		10.75 ± 0.7^a^	8.51–14.80	10.22	3.05	0.19
	2020		0.43**		10.05 ± 0.6^b^	8.48–13.22	7.43	2.07	0.44
Iron (mg/kg)	Pooled	1631.43**	3.23**	3.41**	34.89 ± 1.7	29.23–40.98	6.65	7.13	0.39
	2019		8.22**		38.61 ± 1.9^a^	32.35–46.08	5.95	6.37	0.61
	2020		6.06**		31.18 ± 1.8^b^	25.14–37.91	7.51	6.50	0.52
Zinc (mg/kg)	Pooled	21.74**	2.78**	1.91**	29.27 ± 1.6	24.14–35.68	6.49	5.28	0.50
	2019		5.90**		29.59 ± 1.8^a^	22.44–36.50	6.60	5.43	0.61
	2020		4.14**		28.95 ± 1.6^b^	24.75–34.86	5.33	4.28	0.64

SD, standard deviation; CV, co-efficient of variation; LSD, least significant difference; h^2^, heritability in broad sense. *, **Significant at 0.05, 0.01 probability levels, respectively. The mean followed by the same letters is not significant at p ≤ 0.05, and the mean followed by different letters is significant at p ≤ 0.05.

### Variability Parameters and Heritability (h^2^)

**FIGURE 2:**
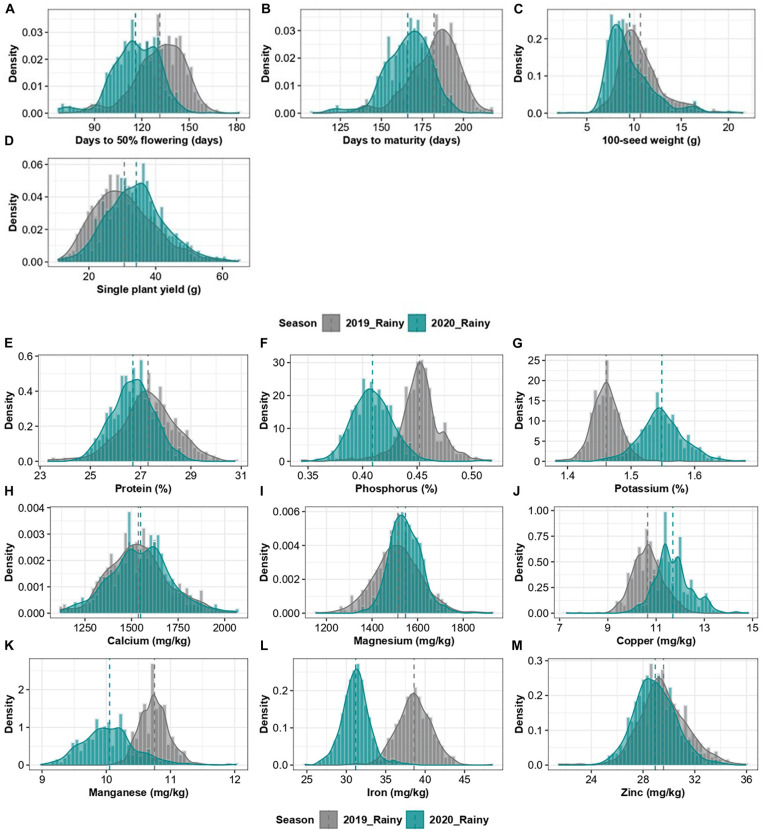
Combined histogram and a density graph, depicting the density of agronomic traits **(A–D)** and grain nutrients **(E–M)** of 2019 and 2020 rainy season crops.

The comparison of mean values of 598 pigeonpea accessions between two cropping years revealed a significant difference in the performance of the accessions for all the traits except for Ca (Table 1). Phenologically, the accessions flowered and matured earlier in the 2020 rainy season (116 ± 14.8 and 166 ± 14.5 days, respectively) compared to the 2019 rainy season (131 ± 16.5 and 182 ± 15.4 days, respectively; Figures 2A,B). Concerning agronomic performance, 100-seed weight was relatively higher in the 2019 rainy crop (10.67 ± 3.0 g) compared to the 2020 rainy crop (9.52 ± 2.6 g; Figure 2C), and grain yield per plant was higher in the 2020 rainy season crop (34.10 ± 7.7 g) compared with 2019 rainy crop (30.63 ± 7.6 g; Figure 2D). For grain nutrients, the 2019 rainy crop had high protein (27.30 ± 1.2%), P (0.45 ± 0.02%), Mn (10.75 ± 0.7 mg/kg), Fe (38.61 ± 1.9 mg/kg), and Zn (29.59 ± 1.8 mg/kg; Figures 2E,F,K,L,M), whereas the 2020 rainy crop had high K (1.55 ± 0.02%), Mg (1546.66 ± 77.6 mg/kg), and Cu (11.69 ± 0.8 mg/kg; Figures 2G,I,J). However, the Ca content (1537.58 ± 173.1 mg/kg and 1548.03 ± 170.3 mg/kg in 2019 and 2020 rainy seasons, respectively) observed no significant difference between the cropping years (Figure 2H). The heritability estimates in broad sense were found to be moderate to high (0.31–0.89 and 0.44–0.99 for the 2019 and 2020 rainy seasons, respectively) for all agronomic and grain nutritional traits in both the cropping years except for K (0.26) and Mn (0.19) in the 2019 rainy season.

The pooled analysis over cropping years presented that the average days to 50% flowering of 598 pigeonpea accession as 124 days encompassing 2-fold variation (67–166 days) and 275 accessions were found to be earlier than the trial mean (Table 1). Similarly, the days to maturity varied from 112 to 213 days with 261 accessions maturing earlier than the trial mean of 174 days. The accessions had wide variability for 100-seed weight, holding very small seeds (1.69 g) to large seeds (22.17 g), with an average 100-seed weight of 10.10 g and 236 accessions surpassed the trial mean. Grain yield per plant varied from 16.54 to 57.93 g, and 271 accessions yielded higher than the trial mean (32.37 g) and ICP 15241 recorded the highest grain yield per plant (57.93 g). For grain nutrients, the accessions varied from 23.35 - 29.50% for protein, 0.36–0.50% for P, 1.43–1.63% for K, 1,311.01–1,865.65 mg/kg for Mg, 8.56–14.01 mg/kg for Mn, 29.23–40.98 mg/kg for Fe, and 24.14–35.68 mg/kg for Zn. With regard to the superiority of the germplasms to the trial mean, 294 accessions for protein (>26.99%), 223 accessions for P (>0.43%), 291 accessions for K (>1.50%), 290 accessions for Mg (>1,530.20 mg/kg), 288 accessions for Mn (>10.40 mg/kg), 291 accessions for Fe (>34.89 mg/kg), and 281 accessions for Zn (>29.27 mg/kg) were found to surpass the trial mean. However, a 2-fold variation was observed for Ca (1,042.36–2,099.76 mg/kg) and Cu (7.72–14.2 mg/kg), with 290 and 291 accessions exceeding the trial mean (1,542.80 mg/kg and 11.17 mg/kg, respectively). High heritability was observed for all agronomic traits except grain yield per plant (0.48). The heritability for grain nutrients was high for Ca (0.70), Mg (0.63), and Cu (0.63), whereas it was moderate for protein (0.31), K (0.33), Mn (0.45), Fe (0.39), and Zn (0.50). Grain phosphorus content had low heritability (0.25).

### Mean Comparison Between Geographical Regions and Maturity Groups

**FIGURE 3:**
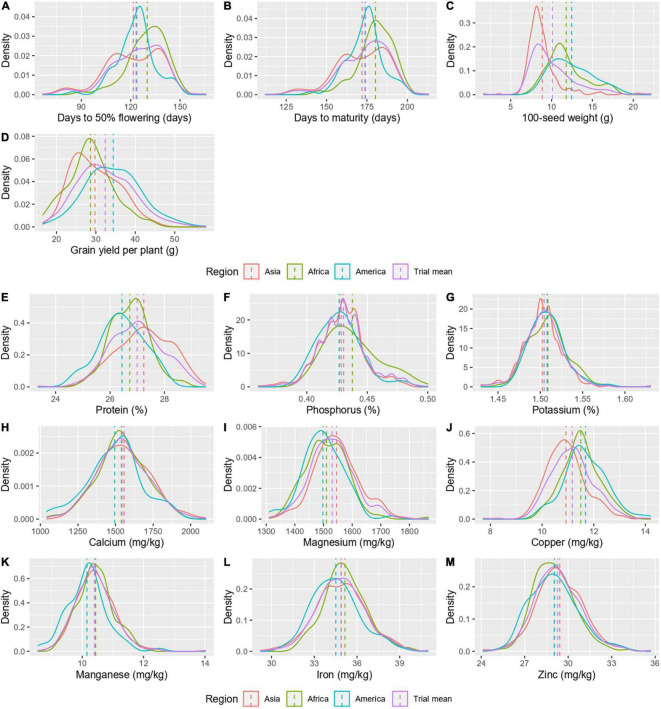
A density graph depicting the distribution of agronomic traits **(A–D)** and grain nutrients **(E–M)** in different geographical regions.

Accessions from three regions, namely, Asia (358), Africa (148), and America (79) were considered for mean comparison, while other regions with few accessions (Europe-11 and Oceania-2) were excluded. The region-wise mean comparison revealed that all agronomic and grain nutritional traits, except for Zn, varied significantly with the geographical region (Table 2). The traits – days to 50% flowering and days to maturity significantly differentiated the Asian (122 ± 16.8 and 172 ± 16.1 days, respectively) and American (123 ± 11.3 and 174 ± 10.8 days, respectively) regions from the African region (130 ± 11.6 and 180 ± 10.9 days, respectively; Figures 3A,B). The 100-seed weight varied significantly in all the three regions, with the American region having a higher 100-seed weight (12.47 ± 2.5 g), followed by the African (11.80 ± 2.4 g) and Asian regions (8.87 ± 2.2 g; Figure 3C). No significant difference was observed between the African and American regions for grain yield per plant; however, the Asian region had a significantly higher yield (34.41 ± 7.1 g; Figure 3D). Protein was the only nutrient to differentiate all the three geographical regions, while other nutrients differentiated one of the three regions. Mean protein content was significantly higher in the Asian region (27.24 ± 1.%), which was followed by the African region (26.73 ± 0.7%) and the American region (26.44 ± 0.8%; Figure 3E). For other nutrients, one region stayed significantly distinct from the other two regions for the P (0.44 ± 0.02%) African region, the K (1.50 ± 0.02%), Mg (1,545.66 ± 77.7 mg/kg), and Cu (10.92 ± 0.8 mg/kg) Asian region, and the Ca (1,494.7 ± 181.2 mg/kg) and Mn (10.16 ± 0.7 mg/kg) American region (Figures 3F–K). For mean Fe content, a significant difference existed between the American (34.51 ± 1.6 mg/kg) and African regions (35.15 ± 1.5 mg/kg), while the Asian region (34.88 ± 1.8 mg/kg) was indifferentiable from the two regions (Figure 3L). For Zn, there was no significant difference between the geographical regions (Figure 3M). The variances remained heterogeneous for all agronomic traits and nutrients, namely, protein, P, and Fe (Table 2).

**TABLE 2:** Mean and range for agronomic and grain nutritional traits of 598 pigeonpea accessions belonging to different regions and maturity groups evaluated during the 2019 and 2020 rainy seasons at ICRISAT, Hyderabad, India.

Trait	Region							Maturity group						
	Mean ± SD			Range			Homo- geneity of variance (*F* value)	Mean ± SD			Range			Homo- geneity of variance (*F* value)
	Asia (358)	Africa (148)	America (79)	Asia (358)	Africa (148)	America (79)		Early (32)	Medium (234)	Late (332)	Early (32)	Medium (234)	Late (332)	
DFF	122 ± 16.8^b^	130 ± 11.6^a^	123 ± 11.3^b^	67–166	88–152	85–148	22.75*	85 ± 7.3^c^	117 ± 8.7^b^	138 ± 5.6^a^	66–100	96–150	126–166	35.77*
DM	172 ± 16.1^b^	180 ± 10.9^a^	174 ± 10.8^b^	112–213	144–201	140–198	22.61*	137 ± 7.6^c^	168 ± 8.2^b^	188 ± 5.4^a^	112–150	151–180	180–213	43.46*
SW (g)	8.87 ± 2.2^c^	11.8 ± 2.4^b^	12.47 ± 2.5^a^	1.69–22.17	7.63–17.78	8.19–18.25	11.17*	8.57 ± 0.9^b^	10.17 ± 2.8^a^	10.20 ± 2.8^a^	7.02–10.46	1.69–22.17	5.63–18.69	9.41*
GYP (g)	34.41 ± 7.1^a^	29.79 ± 5.9^b^	28.62 ± 5.8^b^	16.54–57.93	17.42–45.74	16.66–43.71	4.15*	25.83 ± 5.6^b^	32.40 ± 6.8^a^	33.21 ± 7.1^a^	16.54–41.12	16.66–57.93	17.42–53.61	1.70
Protein (%)	27.24 ± 1.0^a^	26.73 ± 0.7^b^	26.44 ± 0.8^c^	23.35–29.5	25.03–28.68	24.68–28.04	12.92*	27.23 ± 0.9^a^	26.97 ± 1.0^a^	26.99 ± 1.0^a^	24.68–29.12	23.35–29.5	24.77–29.02	0.62
P (%)	0.43 ± 0.02^b^	0.44 ± 0.02^a^	0.43 ± 0.02^b^	0.36–0.48	0.4–0.5	0.39–0.48	5.71**	0.44 ± 0.02^a^	0.43 ± 0.02^a^	0.43 ± 0.02^a^	0.40–0.46	0.37–0.49	0.36–0.50	0.61
K (%)	1.50 ± 0.02^b^	1.51 ± 0.02^a^	1.51 ± 0.02^a^	1.43–1.63	1.45–1.58	1.47–1.58	0.36	1.51 ± 0.02^a^	1.51 ± 0.02^a^	1.50 ± 0.02^a^	1.44–1.55	1.43–1.63	1.44–1.57	0.23
Ca (mg/kg)	1556.49 ± 171.2^a^	1538.74 ± 160.7^a^	1494.72 ± 181.2^b^	1147.71–2099.76	1081.02–1913.49	1042.36–1881.99	1.07	1536.86 ± 148.9^a^	1543.64 ± 171.7^a^	1542.43 ± 171.5^a^	1195.89–2006.41	1042.36–2099.76	1098.43–1965.28	0.28
Mg (mg/kg)	1545.66 ± 77.7^a^	1510.35 ± 75.2^b^	1498.04 ± 71.4^b^	1327.51–1789.81	1335.08–1865.65	1311.01–1734.69	0.57	1556.89 ± 54.9^a^	1535.4 ± 72.5^ab^	1519.17 ± 86.6^b^	1426.27–1684.17	1327.51–1750.32	1311.01–1865.65	4.49*
Cu (mg/kg)	10.92 ± 0.8^b^	11.50 ± 0.7^a^	11.68 ± 0.8^a^	7.72–13.96	9.86–13.4	9.92–14.2	0.94	11.80 ± 0.9^a^	11.24 ± 0.8^b^	10.99 ± 0.8^b^	10.5–13.96	9.43–14.2	7.72–13.4	0.70
Mn (mg/kg)	10.44 ± 10.4^a^	10.43 ± 0.6^a^	10.16 ± 0.7^b^	8.89–14.01	9.19–12.05	8.56–12.51	0.40	10.24 ± 0.4^a^	10.39 ± 0.6^a^	10.43 ± 0.7^a^	9.41–11.25	8.56–14.01	8.89–12.51	2.12
Fe (mg/kg)	34.88 ± 1.8^ab^	35.15 ± 1.5^a^	34.51 ± 1.6^b^	29.23–40.98	31.96–39.69	31.00–39.08	3.40*	36.43 ± 1.7^a^	35.05 ± 1.6^b^	34.47 ± 1.7^c^	33.62–39.81	30.00–39.69	29.23–40.98	0.41
Zn (mg/kg)	29.42 ± 1.6^a^	29.04 ± 1.4^a^	29.04 ± 1.6^a^	24.14–35.68	25.33–34.18	25.51–33.78	1.80	30.99 ± 1.7^a^	29.40 ± 1.5^b^	28.84 ± 1.5^c^	27.46–33.93	25.51–35.68	24.14–33.59	0.90

The value inside the parenthesis represents the number of accessions in each category. SD, standard deviation; DFF, days to 50% flowering; DM, days to maturity; SW, 100-seed weight; GYP, grain yield per plant; P, phosphorus; K, potassium; Ca, calcium; Cu, copper; Mg, magnesium; Mn, manganese; Fe, iron; Zn, zinc. The mean followed by the same letters is not significant at p ≤ 0.05, and the mean followed by different letters is significant at p ≤ 0.05. *Homogeneity of variance tested by Levene’s test is significant at p ≤ 0.05.

**FIGURE 4:**
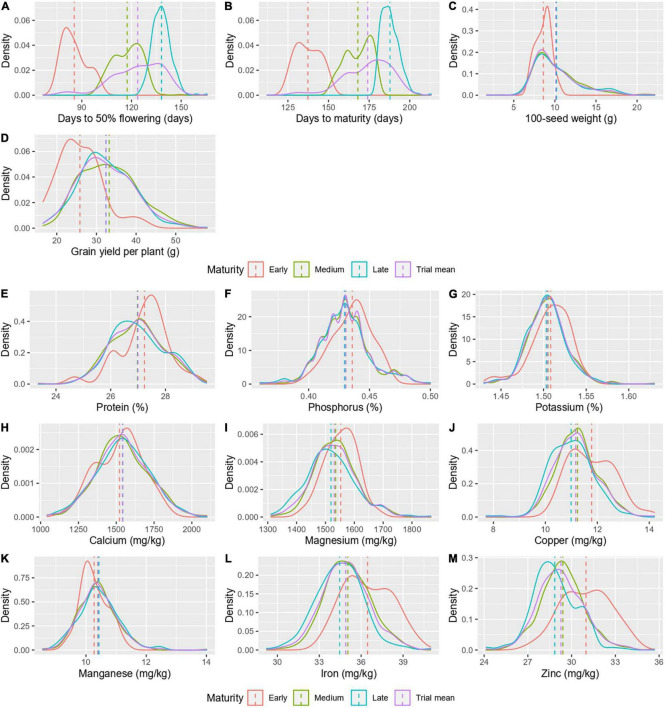
A density graph depicting the distribution of agronomic traits **(A–D)** and grain nutrients **(E–M)** in different maturity groups.

A comparison of agronomic traits and grain nutrients was made between maturity groups, early (32 accessions), medium (234 accessions), and late (332 accessions; Table 2). The mean days to 50% flowering and days to maturity significantly differentiated the maturity groups as the classification was based on the same (Figures 4A,B). Among the three maturity groups, the medium and late maturity groups showed no significant difference for 100-seed weight and grain yield per plant but were significantly higher than the early maturity group (Figures 4C,D). The nutrients, namely, protein, P, K, Ca, and Mn did not vary significantly between the maturity groups (Figures 4E–H,K). However, the mean Fe and Zn content in grain marked a significant difference between the maturity groups, with the early maturity group with high Fe (36.43 ± 1.7 mg/kg) and Zn (30.99 ± 1.7 mg/kg), followed by the medium duration group with intermediate Fe (35.05 ± 1.6 mg/kg) and Zn (29.40 ± 1.5 mg/kg) and the late maturity group with low Fe (34.47 ± 1.7 mg/kg) and Zn content (28.84 ± 1.5 mg/kg; Figures 4L,M). For Mg, the early (1,556.89 ± 54.9 mg/kg) and late maturity group (1,519.17 ± 86.6 mg/kg) varied significantly, while the medium duration group (1,535.40 ± 72.5 mg/kg) was indifferentiable between the two groups (Figure 4I). The Cu content in the early maturity group (11.80 ± 0.9 mg/kg) was high and varied significantly from the other two groups (Figure 4J). The variances were homogenous for all-grain nutrients except for Mg. Agronomic traits had heterogeneous variance except for grain yield per plant.

### Correlation Between Agronomic Traits and Grain Nutrients

**FIGURE 5:**
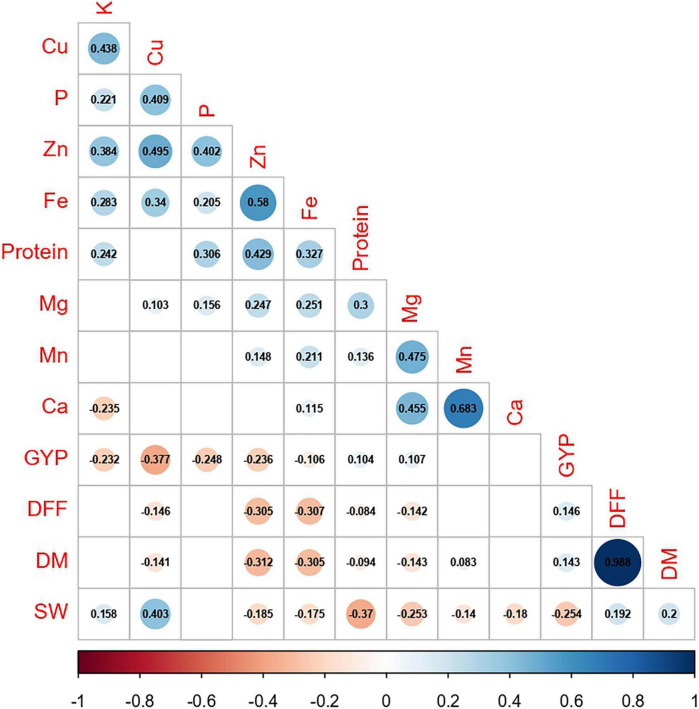
Correlation between agronomic traits and grain nutrients pooled over two cropping years (DFF, days to 50% flowering; DM, days to maturity; SW, 100-seed weight; GYP, grain yield per plant; P, phosphorus; K, potassium; Ca, calcium; Cu, copper; Mg, magnesium; Mn, manganese. Fe, iron; Zn, zinc, respectively. The values represent the significance at *p* ≤ 0.05; blanks represent insignificance at *p* ≤ 0.05).

Among the agronomic traits, a highly significant and positive correlation was seen, except for a significant negative correlation between 100-seed weight and grain yield per plant (*r* = −0.254, *p* ≤ 0.01; Figure 5 and Supplementary Table 2). Protein, the nutrient of great significance in legumes, mostly had a significant positive correlation with all nutrients (*r* = 0.136–0.429, *p* ≤ 0.01), except for a non-significant negative correlation with Ca (*r* = −0.018 and Cu (*r* = −0.024). The correlation between P and K was positive and highly significant (*r* = 0.221, *p* ≤ 0.01), and similar correlations with nutrients, namely, protein, Cu, Fe, and Zn were seen. In addition, P, with a highly significant positive association with Mg (*r* = 0.156, *p* ≤ 0.01), and K, with a highly significant negative association with Ca (*r* = −0.235, *p* ≤ 0.01), were recorded. While the association of Ca was highly significant and positive with Mg, Mn, and Fe (*r* = 0.115–0.683, *p* ≤ 0.01) and mostly non-significant with all other nutrients. Between Fe and Zn existed a highly significant and positive correlation (*r* = 0.580, *p* ≤ 0.01). Other than the correlation for Fe with Ca, Fe (*r* = 0.205–0.340, *p* ≤ 0.01) and Zn (*r* = 0.148–0.495, *p* ≤ 0.01) had a highly significant positive correlation with all other nutrients. The association of days to 50% flowering and days to maturity with grain nutrients was mostly negative and was significant for protein, Mg, Cu, Fe, and Zn. However, Mn recorded a significant positive association with days to maturity (*r* = 0.083, *p* ≤ 0.05). Although, 100-seed weight recorded a highly significant and positive correlation with K (*r* = 0.158, *p* ≤ 0.01) and Cu (*r* = 0.403, *p* ≤ 0.01), the association with most of the nutrients (protein, Ca, Mg, Mn, Fe, and Zn) was found to be negative and highly significant (*r* = −0.140 to −0.370, *p* ≤ 0.01). Between grain yield per plant and nutrients namely, protein and Mg, a significant positive correlation was seen (*r* = 0.104 and 0.107, respectively, *p* ≤ 0.05). On the other hand, the association of grain yield per plant with most of the nutrients, namely, P, K, Cu, Fe, and Zn was negative and highly significant (*r* = −0.106 to −0.377, *p* ≤ 0.01). Withal, Ca recorded no significant correlation with most of the agronomic traits, except 100-seed weight.

A correlation study was conducted in three major geographical regions (Asia, Africa, and America) to identify significant and unique correlations existing between grain nutrients and agronomic traits in each region (Supplementary Tables 3–5). Across all the regions, the association among grain nutrients protein, Ca, Fe, and Zn remains unaltered from the general correlation, except for a non-significant association between Fe and Ca. In the Asian region, days to 50% flowering and days to maturity were significantly positively correlated with each other and with grain yield per plant (*r* = 0.333 and 0.332, *p* ≤ 0.01), whereas, in the African region, was instead with 100-seed weight (*r* = 0.458 and 0.469, *p* ≤ 0.01). Furthermore, 100-seed weight with a significant negative correlation with grain yield per plant (*r* = −0.325, *p* ≤ 0.01) was observed only in the African region, and, in other regions, it was insignificant. In the American region, a significant positive correlation existed only between days to 50% flowering and days to maturity (*r* = 0.985, *p* ≤ 0.01). Between agronomic traits and grain nutrients, namely, protein and Ca, the association was non-significant in all the three regions, except for a significant negative association with 100-seed weight in the Asian (*r* = −0.292 and *r* = −0.173, respectively, *p* ≤ 0.01) and American regions (*r* = −0.304 and *r* = −0.285, respectively, *p* ≤ 0.01). Concerning Fe and Zn, the association was significant and negative with all the agronomic traits in the Asian region. In the African and American regions, the association of Fe and Zn with most of the agronomic traits was negative and significant, except for a non-significant negative association for Fe with grain yield per plant and Zn with 100-seed weight.

As the Fe and Zn content varied with the maturity group, the correlation study was conducted in each maturity group (Supplementary Tables 6–8). The association of the protein with days to 50% flowering, days to maturity, and 100-seed weight was non-significant in the early maturity group, significant and negative in the medium maturity group (*r* = −0.221 to −0.439, *p* ≤ 0.01), and non-significant in the late maturity group, except for a significant negative association with 100-seed weight (*r* = −0.288, *p* ≤ 0.01). Protein was significantly positively correlated with grain yield per plant in the early (*r* = 0.392, *p* ≤ 0.05) and late maturity groups (*r* = 0.125, *p* ≤ 0.05) and was non-significant in the medium maturity group. Across all the maturity groups, Ca was significantly negatively correlated with 100-seed weight (*r* = −0.165 to −0.452, *p* ≤ 0.01) and non-significantly with all other agronomic traits, except for a significant positive association with days to maturity (*r* = 0.143, *p* ≤ 0.05) in the late maturity group. In the early maturity group, Fe and Zn had a non-significant association with all agronomic traits, except for a significant negative association between Zn and grain yield per plant (*r* = −0.493, *p* ≤ 0.01). In the medium maturity group, the association of Fe and Zn with all agronomic traits was negative and significant. In the late maturity group, Fe was significantly negatively correlated with days to maturity (*r* = −0.143, *p* ≤ 0.05), whereas Zn had a significant positive association with days to 50% flowering (*r* = 0.137, *p* ≤ 0.05) and a significant negative association with 100-seed weight (*r* = −0.182, *p* ≤ 0.01).

The 100-seed weight marks the consumer preference, and it is noteworthy to study its association with grain nutrients (Supplementary Tables 9–11). Protein had a significant negative correlation with 100-seed weight of ≤ 10 g (*r* = −0.156, *p* ≤ 0.01) and 10–15 g (*r* = −0.222, *p* ≤ 0.01), and was non-significant beyond 15 g. With ≤ 10-g 100-seed weight, the grain Ca content was significantly negatively correlated (*r* = −0.322, *p* ≤ 0.01) beyond which there was no significant correlation. Concerning Fe and Zn, mostly existed a non-significant association with 100-seed weight, except for a significant negative association with Fe (*r* = −0.329, *p* ≤ 0.05) for >15-g 100-seed weight.

### Cluster Analysis

**FIGURE 6:**
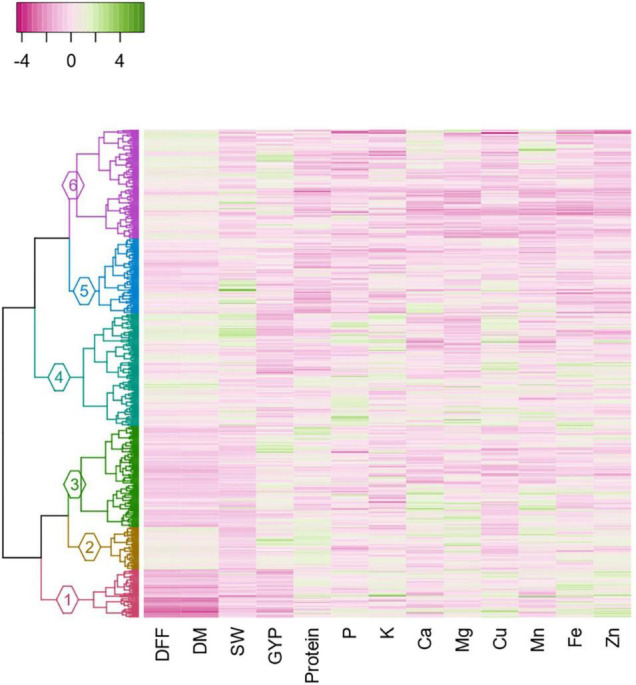
Dendrogram constructed based on the Gower’s distance matrix, adopting Ward. D2 clustering method with heatmap depicting the agronomic and grain nutrient content in each accession of the cluster (DFF, days to 50% flowering; DM, days to maturity; SW, 100-seed weight; GYP, grain yield per plant; P, phosphorus; K, potassium; Ca, calcium; Cu, copper; Mg, magnesium; Mn, manganese; Fe, iron; Zn, zinc).

**FIGURE 7:**
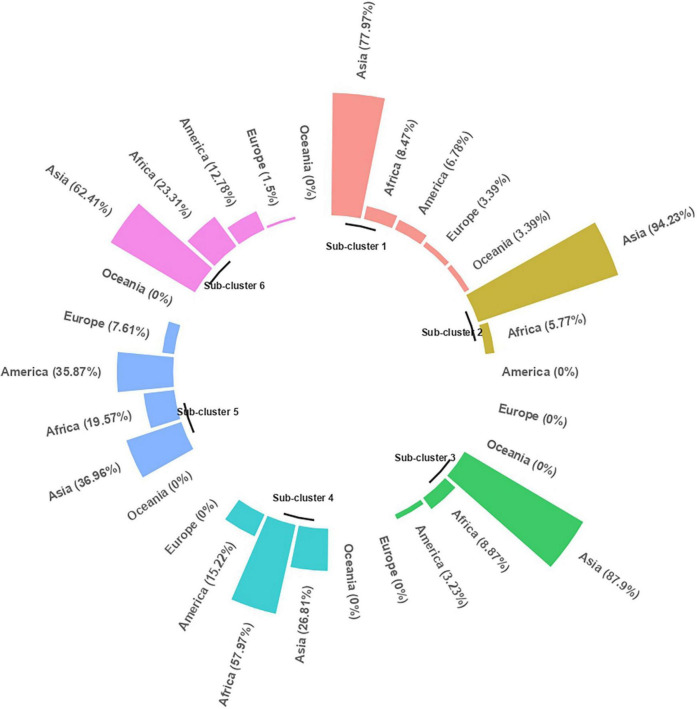
Sub-clusters with percent geographical distribution.

The clustering based on Gower’s distance matrix apportioned the 598 pigeonpea accessions into two major clusters with three sub-clusters each at h = 0.7 (Figure 6 and Supplementary Figure 1). The minimum and maximum numbers of accessions were observed in sub-clusters 2 (52) and 4 (138), respectively. The other sub-clusters 1, 3, 5, and 6 had 59, 124, 92, and 133 accessions, respectively (Supplementary Table 12). The region-wise contribution identified major Cluster I, with accessions predominantly from the Asian region, whereas the major Cluster II with the co-clustering of accessions from all regions (Figure 7 and Supplementary Figure 1). Despite the domination of the Asian region in the major Cluster 1, the Sub-cluster 1 had accessions from all other regions (<10%). Within the major Cluster 2, Sub-cluster 6 had 62.41% accessions from the Asian region, along with 23.31, 12.78, and 1.50% accessions from African, American, and European regions, respectively. The co-clustering of accessions from different regions was predominantly found in Sub-clusters 4 and 5. The Sub-cluster 4 had 57.97, 26.81, and 15.22% accessions from the African, Asian, and American regions, respectively. In Subcluster 5, the Asian, African, American, and European regions contributed 36.96, 19.57, 35.87, and 7.61%, respectively. The genetic similarity/dissimilarity among accessions between and within sub-clusters was determined by inter and intra-cluster distances. The intra-cluster distance identified Sub-cluster 1 (*d* = 0.136) as the more diverse sub-cluster with maximum intra-cluster distance and Sub-cluster 2 with the least (*d* = 0.099; Supplementary Table 13). Similarly, the maximum inter-cluster distance was observed between Sub-clusters 1 and 6 (*d* = 0.227), followed by Sub-cluster 1 with Sub-clusters 2 and 4 (*d* = 0.187). Overall, Sub-cluster 1 had the maximum inter-cluster distance with all other sub-clusters. The least inter-cluster distance was observed between Sub-clusters 2 and 3 and, Subclusters 5 and 6 (*d* = 0.143).

The distribution of each grain nutrient and its corresponding agronomic performance in each sub-cluster is displayed in the heatmap, with a varying intensity of pink (low) to green (high) color, which characterizes the sub-cluster (Figure 6). The mean comparison between sub-clusters revealed that the sub-clusters varied significantly from each other for all agronomic traits and grain nutrients (Supplementary Table 14). Days to 50% flowering and days to maturity distinguished 4 out of 6 sub-clusters, with Sub-cluster 1 being the earliest in flowering, followed by Sub-clusters 3 and 5. The Sub-clusters 2, 4, and 6 were insignificantly different from each other for both the traits. Sub-clusters 4 and 5 had high 100-seed weight, whereas Sub-clusters 2, 3, and 6 had high grain yield per plant. Protein, Fe, and Zn distinguished five out of six sub-clusters. For specific nutrient sources, Sub-cluster 2 contained protein-dense accessions (28.15 ± 0.6%), Sub-cluster 3 for Ca (1583.83 ± 194.3 mg/kg), Sub-cluster 1 for Fe (36.62 ± 1.5 mg/kg) and Zn (31.21 ± 1.4 mg/kg). However, the Ca content in the Sub-clusters 1, 2, 4, and 5 was found to be indifferentiable from Sub-clusters 3 and 6. For other nutrients, the nutrient-dense accessions were found in Sub-clusters 1 and 4 for K and Cu, Subcluster 4 for P, and Sub-cluster 2 for Mg and Mn. Overall, high mean for four nutrients (Fe, Zn, K, and Cu) was observed in Sub-cluster 1 and for 3 nutrients in Sub-clusters 2 (Protein, Mg, and Mn) and 4 (P, K, and Cu).

### Nutrient-Dense Accessions

Accessions with high nutrient density were identified based on the superiority to the trial mean and the superior check. Among the two checks, check ICP 8863 was found to have better nutrient content with 27.69% protein, 0.44% P, 1.51% K, 1,497.59 mg/kg Ca, 1,630.96 mg/kg Mg, 11.14 mg/kg Cu, 10.62 mg/kg Mn, 38.19 mg/kg Fe, and 32.58 mg/kg Zn. The number of superior accessions was 139 for protein, 107 for P, 171 for K, 290 for Ca, 53 for Mg, 291 for Cu, 197 for Mn, 21 for Fe, and 16 for Zn. The top 10 nutrient-specific accessions covered a range of 28.85–29.50% protein, 38.67–40.98 mg/kg Fe, 32.97–35.68 mg/kg Zn, and 1,923.79–2,099.76 mg/kg Ca. For other nutrients, the ranges were 0.48–0.49% for P, 1.55–1.58% for K, 1,710.14–1,865.65 mg/kg for Mg, 13.08–13.96 mg/kg for Cu, and 11.79–12.51 mg/kg for Mn. The multi-nutrient dense accessions were screened from the top 10 nutrient-specific accessions identified for each nutrient (Table 3). Fifteen accessions, representing eight countries and three geographical regions, were identified as superior sources for 3–7 nutrients (Table 4). Of these, eight out of 10 accessions in the Asian region are from India. These 15 accessions varied widely for days to 50% flowering and maturity (77–144 and 127–192 days, respectively). Among these, four accessions for 100-seed weight and six accessions for grain yield per plant were superior to the trial mean and check ICP 8863. However, the yield of these accessions (16.54–45.53 g) was not superior to the check ICP 7221 (48.93 g). These 15 accessions belonged to four sub-clusters (sub-clusters 1, 2, 3, and 4). Among the 15 accessions, ICP 7533 was identified as the best source for seven nutrients, followed by accessions ICP 8165, ICP 11485, ICP 12043, and ICP 13757 for six nutrients.

**TABLE 3:** Top ten pigeonpea accessions with high trait value identified from 598 pigeonpea accessions evaluated during the 2019 and 2020 rainy seasons at ICRISAT, India.

Trait	Trial mean	Mean+LSD_0.05_	Top 10 accession (ICP#)	Nutrient range	DFF (days)	DM (days)	SW (g)	GYP (g)
Protein (%)	26.99	32.16	6027, 5369, 6165, 15249, 15247, 2860, 5960, 15237, 6219, 8165	28.85–29.50	110–140	159–188	7.85–9.06	29.3–52.70
P (%)	0.43	0.58	12788, 13046, 9185, 11350, 12064, 12043, 11863, 10086, 12828, 12832	0.48–0.49	105–141	155–187	6.76–14.69	21.62–41.64
K (%)	1.50	1.72	11485, 15109, 13315, 11350, 15106, 14109, 8035, 7399, 7028, 13757	1.55–1.58	81–144	135–192	7.18–14.36	19.35–33.79
Ca (mg/kg)	1542.8	2019.63	**7867, 8354**, 15597, 8392, 11472, 1458, 10298, 1514, 774, 10876	1923.79–2099.76	67–142	112–193	6.53–16.01	22.18–43.76
Mg (mg/kg)	1530.2	1748.62	**12043, 15269, 8588, 1514, 7439,** 13553, 7337, 13994, 11991, 12558	1710.14–1865.65	109–159	160–205	6.91–12.46	25.01–44.04
Cu (mg/kg)	11.17	13.26	**7533, 13857, 9146, 13807,** 12928, 13315, 14389, 7028, 15489, 13551	13.08–13.96	77–144	127–193	7.99–20.35	16.54–29.62
Mn (mg/kg)	10.40	12.58	13545, 14574, 12538, 8354, 12174, 9185, 7870, 7982, 11849, 7867	11.79–12.51	103–148	155–198	7.00–16.01	27.27–43.76
Fe (mg/kg)	34.89	42.02	10876, 9123, 13576, 7650, 8165, 1050, 13551, 8392, 8042, 7347	38.67–40.98	79–138	130–188	7.02–9.83	19.56–45.53
Zn (mg/kg)	29.27	34.55	**11485,** 16844, 14600, 7533, 7903, 11350, 8015, 8356, 6219, 13757	32.97–35.68	77–138	127–188	7.19–17.42	16.54–43.07

*DFF, days to 50% flowering; DM, days to maturity; SW, 100-seed weight; GYP, grain yield per plant; P, phosphorus; K, potassium; Ca, calcium; Cu, copper; Mg, magnesium; Mn, manganese; Fe, iron; Zn, zinc. Bolded accession number represents superiority to trial mean + LSD_0.05_.*

**TABLE 4:** Promising multi-nutrient dense pigeonpea landraces identified from 598 pigeonpea accessions evaluated during the 2019 and 2020 rainy seasons at ICRISAT, India.

S.No	Accession	Country	Maturity group	Sub-cluster	DFF (days)	DM (days)	SW (g)	GYP (g)	Protein (%)	P (mg/kg)	K (mg/kg)	Ca (mg/kg)	Mg (mg/kg)	Cu (mg/kg)	Mn (mg/kg)	Fe (mg/kg)	Zn (mg/kg)
**Asia**																	
1	ICP 6219	India	Medium	3	122	171	8.12	**43.07**	**28.95**	**0.45**	1.50	1440.92	1521.33	**11.97**	10.38	36.62	**32.99**
2	ICP 8165	India	Late	2	138	188	9.06	**45.53**	**28.85**	0.43	**1.52**	**1657.89**	1565.65	**11.60**	**11.66**	**39.18**	31.00
3	ICP 7867	India	Medium	3	127	179	**16.01**	**43.76**	26.37	0.40	1.49	**2099.76**	**1654.93**	10.81	**11.79**	33.85	27.84
4	ICP 1514	India	Medium	3	111	160	7.4	**41.25**	26.83	0.42	1.47	**1955.06**	**1750.32**	10.84	**11.68**	34.34	27.80
5	ICP 7028	India	Early	1	81	135	9.5	19.35	27.13	**0.46**	**1.55**	1495.66	1552.74	**13.09**	10.28	35.36	31.14
6	ICP 7533	India	Early	1	77	127	7.99	16.54	27.26	**0.46**	**1.53**	**1672.91**	**1614.33**	**13.96**	**10.90**	38.17	**33.76**
7	ICP 8354	India	Medium	3	104	155	8.32	**35.13**	27.00	0.41	1.49	**2049.67**	1567.97	**11.23**	**12.08**	37.08	31.45
8	ICP 8392	India	Medium	3	111	163	7.74	**41.57**	**28.03**	0.42	1.51	**1968.65**	1523.76	**11.59**	**11.61**	**39.00**	32.54
9	ICP 11350	Nepal	Late	4	138	188	9.2	28.38	**28.65**	**0.48**	**1.56**	1293.94	1558.29	**11.98**	9.89	35.79	**33.59**
10	ICP 11485	Thailand	Medium	1	103	156	7.37	20.34	**28.63**	**0.47**	**1.58**	1188.95	1505.61	**12.35**	9.35	**38.57**	**35.68**
**Africa**																	
11	ICP 9185	Kenya	Medium	4	129	179	**12.91**	27.27	26.89	**0.49**	1.51	**1716.81**	1587.93	**11.69**	**12.05**	35.21	31.23
12	ICP 12043	Tanzania	Late	4	134	187	**10.89**	28.58	**28.13**	**0.48**	1.51	**1751.98**	**1865.65**	**11.37**	**11.74**	33.58	29.89
13	ICP 13315	Rwanda	Late	4	144	192	**11.48**	23.23	**28.66**	**0.46**	**1.57**	1205.61	1439.64	**13.14**	9.19	34.95	30.12
**America**																	
14	ICP 13757	Trinidad and Tobago	Medium	4	129	179	10.07	30.64	**28.04**	**0.45**	**1.55**	**1594.69**	1504.33	**12.39**	10.24	35.90	**32.97**
15	ICP 13551	Antigua and Barbuda	Medium	4	126	177	8.71	19.56	27.08	**0.44**	1.51	1448.73	1599.75	**13.08**	9.58	**39.08**	**32.87**
Minimum					77	127	7.37	16.54	26.37	0.40	1.47	1188.95	1439.64	10.81	9.19	33.58	27.8
Maximum					144	192	16.01	45.53	28.95	0.49	1.58	2099.76	1865.65	13.96	12.08	39.18	35.68

*DFF, days to 50% flowering; DM, days to maturity; SW, 100-seed weight; GYP, grain yield per plant; P, phosphorus; K, potassium; Ca, calcium; Cu, copper; Mg, magnesium; Mn, manganese; Fe, iron; Zn, zinc. Bold values indicate the superiority over the trial mean and superior check.*

## Discussion

Between germplasm availability and its subsequent utilization in crop improvement programs, there exists a huge gap. The attributable reasons are i) a lack of information about the genetic worth of the germplasm, ii) presence of undesirable linkages, difficulties, and expensiveness linked in screening for few elite lines from a vast ocean of germplasm, iii) risk of crossing program failure and the long time scale linked in the development of breeding lines, and iv) the possibility of toxins and allergens introduction into the elite cultivars during introgression (Upadhyaya et al., 2010; Mallikarjuna et al., 2014). Pigeonpea offers an affordable source of protein to the marginalized populations surviving in several developing countries of Asia and Africa. Other than protein, pigeonpea is rich in a few minerals too, and, more interestingly, the accumulation of Fe and Zn in the cotyledons benefits by overcoming the dehulling nutrient loss, which is common in cereals like wheat and rice (Susmitha et al., 2022). Identification of nutrient-rich germplasm can further enrich the breeders’ crossing blocks for developing high-yielding and nutrient-rich varieties.

The REML analysis indicated the existence of adequate variability in the germplasm for all agronomic traits and grain nutrients. Other than Ca, the variance attributable to the environment was significant for all the traits, indicating that the extraneous factors contained in the cropping years were different and adequate in differentiating the accessions. The significant G × E interaction for most of the traits indicated the sensitivity of nutrients accumulation to the environment. This suggests for further evaluation of the germplasm in multiple locations and multiple years to have a better insight into the G × E interaction existing for the traits (Murube et al., 2021) and selection thereafter. Low G × E interaction and moderate-to-high heritability for most of the traits studied suggest a better selection response. The heritability estimates of agronomic traits stay parallel with several studies (Kumara et al., 2013; Rangare et al., 2013; Obala et al., 2018; Shruthi et al., 2019; Sharma et al., 2020), while the estimates for protein content were variable across studies. The attributable reason may be due to the variable number of genotypes and the environment under evaluation (Obala et al., 2018). Wide variability, insensitivity to G × E interaction, and high heritability of Ca identify this nutrient to have stable trait-associated variants in genome-wide association studies (GWAS). The availability of reference genome sequence in pigeonpea (Varshney et al., 2012; Garg et al., 2021) facilitates the application of GWAS to understand the genetic basis of grain nutrient accumulation and to identify candidate genes or genomic regions associated with these nutrients in future studies to breed biofortified pigeonpea cultivars. However, earlier studies pertaining to the association of genomic regions with domestication and agronomic traits were reported (Varshney et al., 2017; Zhao et al., 2020).

Pulses are rich sources of protein, vitamins, and minerals. Combined with relatively low cost and wide access to the poor, pulses are characterized as “poor man’s meat” (Malo and Hore, 2020). The variability observed for whole-grain protein in the present study (23.35–29.50%) was higher than the protein content (16.76–26.82%) reported in previous studies (Amarteifio et al., 2002; Sekhon et al., 2017; Obala, 2018; Cheboi et al., 2019; Choi et al., 2020; Jawalekar et al., 2020) and is comparable with the *dhal* protein content of high protein lines (27–29%; Saxena et al., 1987). The protein content in *dhal* is higher than that in the whole grain (Susmitha et al., 2022), signifying that *dhal* nutritional analysis of the superior accessions in this study may still have higher protein than the high protein lines reported by Saxena et al. (1987). This indicated the availability of superior parental sources for protein biofortification. In specific, the protein content of wild species *Cajanus cajanifolius and C. sericeus* (∼29%) was similar to the previous study (Upadhyaya et al., 2013). On par with wild species, few landraces *viz.* ICP 6027, ICP 5369, ICP 15249, ICP 15247, and ICP 6165 had similar protein content (∼29%) and belonged to medium and late maturity groups. These sources from the primary gene pool can make crossing or gene transfer easy compared to those involving the secondary gene pool (Harlan and de Wet, 1971).

The pigeonpea is found to be rich in calcium (Saxena et al., 2010; Susmitha et al., 2022), and the results of this study inferred that the Ca content in pigeonpea (154. 28 mg/100 g) was found to be higher than many staple cereals (7.49–39.36 mg/100 g), such as rice, wheat, maize, pearl millet, sorghum, and barley but lesser to Ca-dense finger millet (364 mg/100 g). Among grain legumes, pigeonpea stands next to soybean (239 mg/100 g) in whole-grain-Ca content (Longvah et al., 2017). Furthermore, a good amount of K (15,000 mg/kg) and Mg (1,530.20 mg/kg) is accumulated in the pigeonpea whole grain, which can reduce the risk of cardiovascular diseases and diabetes when included in the diet (Schulze et al., 2007; Cherbuin, 2016; Stone et al., 2016). In pigeonpea, the Fe and Zn content in cotyledon is indifferentiable from the whole-grain Fe and Zn (Susmitha et al., 2022). This indicates that the Fe and Zn content reported in this study not only represents the whole grain but also the cotyledon. The Fe content in pigeonpea (3.49 mg/100 g) is low when compared to other pulses like chickpea, black gram, horse gram (5.97–8.76 mg/100 g), while the Zn content (2.93 mg/100 g) is comparable with these pulses (2.71–3.37 mg/100 g; Longvah et al., 2017). This necessitates their subsequent improvement through intra or inter-specific hybridization. To enhance the variability for Fe and Zn in the primary gene pool, Sharma et al. (2020) attempted interspecific crosses with *Cajanus platycarpus*. Despite this, a good response to agronomic biofortification for Fe and Zn was reported in pigeonpea (Gopalakrishnan et al., 2016; Hanumanthappa et al., 2018; Behera et al., 2020). However, Upadhyaya et al. (2010) identified 14 high Zn accessions from core and mini-core collections of pigeonpea available in Genebank at ICRISAT, India. Furthermore, two accessions for Ca (2,049.67–2,099.76 mg/kg), four accessions for Mg (1,750.32–1,865.65 mg/kg), five accessions for Cu (13.34–14.20 mg/kg), and one accession for Zn (35.68 mg/kg), with significantly higher nutrient content than the trial mean identified in this study, enlighten the presence of potential germplasm for mineral biofortification in the ICRISAT Genebank.

The nutrients among themselves were positively correlated with one another, thus facilitating combined multi-nutrient biofortification. The protein improvement in pigeonpea is favored by selection for nutrients, namely, P, K, Mg, Mn, Fe, and Zn. The nutrients, Fe and Zn, are highly positively correlated with each other, and, hence, their improvement together stays significant. This correlation existed across several legumes, such as pigeonpea (Mishra and Acharya, 2018; Sharma et al., 2020), common bean (Celmeli and Sari, 2018), cowpea (Dakora and Belane, 2019), and green gram (Singh et al., 2018). Furthermore, the positive correlation of Fe with all other nutrients offers opportunities for reciprocal nutrient improvement. For Ca improvement, the selection can be done for Mg, Mn, and Fe or against K. This relation stays analogous to the results of Sharma et al. (2020) for the association of Ca with Fe and Mg and Gerrano et al. (2019) for Ca with K.

In recent years, extensive research has been carried out to develop more super-early, extra-early, and early types as photo insensitivity is directly related to earliness, which can break an adaptation barrier and help in the introduction of the crop in new niches and can diversify traditional cereal-based cropping systems (Saxena et al., 2018, 2019). The variability for days to maturity identified the presence of extra-early accession (ICP 15597), a released cultivar (MN1), which has been exploited for breeding high-yielding super-early varieties (Srivastava et al., 2012). Interestingly, the days to 50% flowering and the days to maturity were found to be negatively associated with protein, Mg, Cu, Fe, and Zn, which complements the development of early lines with high nutrient content. However, the pigeonpea cultivated worldwide belongs to medium and late-maturity groups. The presence of indifferentiable Ca and protein content across different maturity groups stands as an advantage for improving Ca and protein in different maturity groups, which can fit into different cropping systems across the globe. Furthermore, Zn exhibited a non-significant association with grain yield per plant among the early- and late-maturity groups, which is of great significance in promoting food security and overcoming Zn deficiency worldwide.

The choice of pigeonpea varieties cultivated across different geographical regions is decided by the market value and/or consumer preference. The seed size defines the consumer preference, and the most preferred seed size in Indian market is 10–14 g/100 seeds (Varshney et al., 2017), whereas, in African and the Caribbean regions, it is about 18 g/100 seeds (Saxena et al., 1987). The mean 100-seed weight (10.10 g) indicated that most of the accessions were distributed around the mean, which is preferable in the Indian market. Forty-two accessions recorded more than 15 g per 100 seeds, of which African and American regions alone contributed 19 and 14 accessions, respectively, reflecting their seed size preferences. The correlation analysis revealed that the nutrient improvement (protein, Ca, Fe, and Zn) in pigeonpea is favored by selection for a small seed size (less 100-seed weight). This can be related to most of the wild species, with small seed size having high nutrient content in pigeonpea. The region-based correlation analysis revealed that protein and Ca improvement in the African region is unaffected by 100-seed weight. Furthermore, the100-seed weight does not affect the improvement of Ca and Zn beyond 10 g, Fe up to 15 g, and protein beyond 15 g. Similar to this, an earlier report on variable association of protein with 100-seed weight in different intergeneric crosses was reported by Saxena et al. (1987).

The yield of majority of the staple crops was stagnated and/or unable to meet global demand. For further genetic improvement, variability for the trait is essential. The grain yield per plant recorded good variability and inhibited a positive correlation with protein and Mg, and a non-significant association with Ca. These useful correlations can be utilized in enhancing the nutrient content along with yield, which can promote combined food and nutritional security. However, the high coefficient of variation observed for the trait is attributed toward the variable number of plants across accessions.

Trading played a key role in the introduction of landraces from India to Africa (Hillocks et al., 2000) and from Africa to America (Van Der Maesen, 1980), which created the possibility for the existence of allochthonous landraces in these regions, which, over time, might have crossed with autochthonous landraces of the region and evolved as autochthonous landraces, sharing some common features between regions (Zeven, 1998), leading to the co-clustering of accessions from different regions within a cluster. Geographical diversity combined with high nutrient density in the Sub-clusters 1, 2, and 4 can provide a valuable parental source for introducing new variability in the primary gene pool of pigeonpea for grain nutrient improvement in different regions. Furthermore, the 10-trait specific and 15 multi-nutrient dense accessions identified based on the *per se* performance and superiority to the nutrient dense check belong to different geographical regions and exhibited wide variation for agronomic traits. These germplasms can be utilized to improve the grain nutrient content under different seed sizes and maturity categories. Furthermore, the pigeonpea breeding community across the globe can get access to the limited quantity of the seed through the Standard Material Transfer Agreement.

## Conclusion

The study revealed the presence of considerable variability and moderate-to-high heritability for the agronomic traits and grain nutrients in the primary gene pool of pigeonpea germplasm. The distribution and the association of grain nutrients among themselves and with agronomic traits were variable across the geographical region and maturity groups, which could benefit the breeders in identification of region- and maturity-group-specific sources and associations, respectively, which can eliminate the risk of acclimatization in the newly breed cultivars. The trait-specific sources identified for grain nutrients content can provide a new parental base in the biofortification program for the development of nutrient-dense cultivars in a desirable agronomic background that can promote food and nutritional security. However, with the available low-cost sequencing technology, genotyping of the 600 accessions in the future, combined with the large phenotypic data generated in this study, can serve as a valuable raw material for conducting SNP/haplotype-based GWAS to identify genetic variants associated with the nutrients that can accelerate genetic gains in pigeonpea biofortification.

## Data Availability Statement

The original contributions presented in this study are included in the article/Supplementary Material, further inquiries can be directed to the corresponding author.

## Author Contributions

KS, RS, and MV to the conception and design of the study (this work is part of DS’s Ph. D. thesis research). TK, VA, and KS supported student research as supervisors. OP provided resources (seed material) for the study. PC and CN performed laboratory analysis. SR supported in data collection and VNA in data documentation. DS, RS, MV, TK, and KS curated the data and performed the formal data analysis. SM, PJ, DS, MV, and BA did data validation and visualization. DS, MV, and KS were involved in writing the original draft, reviewing, and editing. All authors contributed to the article and approved the submitted version.

## Conflict of Interest

The authors declare that the research was conducted in the absence of any commercial or financial relationships that could be construed as a potential conflict of interest.

## Publisher’s Note

All claims expressed in this article are solely those of the authors and do not necessarily represent those of their affiliated organizations, or those of the publisher, the editors and the reviewers. Any product that may be evaluated in this article, or claim that may be made by its manufacturer, is not guaranteed or endorsed by the publisher.

## Footnotes

1
http://genebank.icrisat.org/

## Supplementary Material

Supplementary Figure 1 Dendrogram constructed based on Gower’s distance using Ward. D2 clustering algorithm for 598 pigeonpea accessions.

Supplementary Table 1 Metadata of the 600 pigeonpea accessions and four checks evaluated for agronomic traits and grain nutrients during the 2019 and 2020 rainy seasons at ICRISAT, India.

Supplementary Table 2 Correlation among agronomic traits and grain nutrients of 598 pigeonpea accessions evaluated during the 2019 and 2020 rainy seasons at ICRISAT, India.

Supplementary Table 3 Correlation between agronomic traits and grain nutrients for accessions from Asian region.

Supplementary Table 4 Correlation between agronomic traits and grain nutrients for accessions from African region.

Supplementary Table 5 Correlation between agronomic traits and grain nutrients for accessions from American region.

Supplementary Table 6 Correlation between agronomic traits and grain nutrients for accessions from the early maturity group.

Supplementary Table 7 Correlation between agronomic traits and grain nutrients for accessions from the medium maturity group.

Supplementary Table 8 Correlation between agronomic traits and grain nutrients for accessions from the late maturity group.

Supplementary Table 9 Correlation between agronomic traits and grain nutrients for accessions with ≤ 10 g 100-seed weight.

Supplementary Table 10 Correlation between agronomic traits and grain nutrients for accessions with 10-15 g 100-seed weight.

Supplementary Table 11 Correlation between agronomic traits and grain nutrients for accessions with > 15 g 100-seed weight.

Supplementary Table 12 Contribution of accessions from different regions to each sub-cluster.

Supplementary Table 13 Within and between cluster distances based on Gower’s phenotypic distance matrix for 598 pigeonpea accessions evaluated during the 2019 and 2020 rainy seasons at ICRISAT, India.

Supplementary Table 14 Mean and range comparison for agronomic traits and grain nutrients between six sub-clusters of 598 pigeonpea accessions evaluated during the 2019 and 2020 rainy seasons at ICRISAT, India.
