# Corrigendum: Grain nutrients variability in pigeonpea genebank collection and its potential for promoting nutritional security in dryland ecologies

**DOI:** 10.3389/fpls.2022.1087262

**Published:** 2022-12-12

**Authors:** Dhanapal Susmitha, Thiyagarajan Kalaimagal, Ramachandran Senthil, Mani Vetriventhan, Swaminathan Manonmani, Prabhakaran Jeyakumar, Bellie Anita, Surender Reddymalla, Pushpajeet L. Choudhari, Chetna A. Nimje, Ovais H. Peerzada, Venkata Narayana Arveti, Vania C. R. Azevedo, Kuldeep Singh

**Affiliations:** ^1^ Genebank, International Crops Research Institute for the Semi-Arid Tropics (ICRISAT), Patancheru, India; ^2^ Centre for Plant Breeding and Genetics, Tamil Nadu Agricultural University (TNAU), Coimbatore, India; ^3^ Office of the Registrar, Tamil Nadu Agricultural University (TNAU), Coimbatore, India; ^4^ Directorate of Open Distance Learning, Tamil Nadu Agricultural University (TNAU), Coimbatore, India; ^5^ Charles Renard Analytical Laboratory, International Crops Research Institute for the Semi-Arid Tropics (ICRISAT), Patancheru, India; ^6^ International Potato Center (CIP), Lima, Peru

**Keywords:** pigeonpea, protein, minerals, calcium, biofortification, landraces

In the published article, there was an error in **Figure 2A** as published. The mean line went beyond the range. The corrected **Figure 2** and its caption appear below:

**Figure 2:**
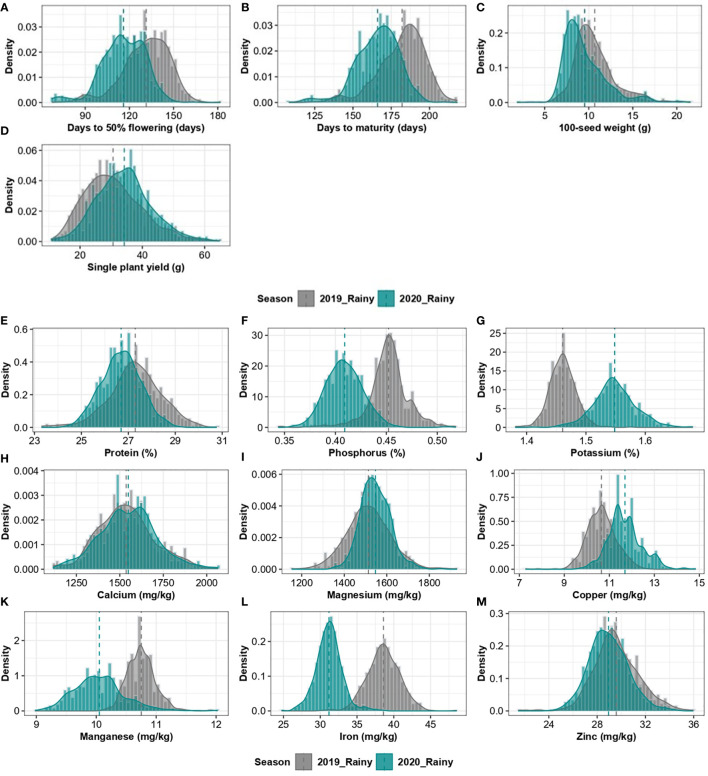
Combined histogram and a density graph, depicting the density of agronomic traits **(A–D)** and grain nutrients **(E–M)** of 2019 and 2020 rainy season crops.

The authors apologize for this error and state that this does not change the scientific conclusions of the article in any way. The original article has been updated.

