# Supplementary figures and images for: Grain Nutrients Variability in Pigeonpea Genebank Collection and Its Potential for Promoting Nutritional Security in Dryland Ecologies
Source: Front Plant Sci. 2022 Jul 11;13:934296. doi: 10.3389/fpls.2022.934296 (PMC9310011; doi:10.3389/fpls.2022.934296)

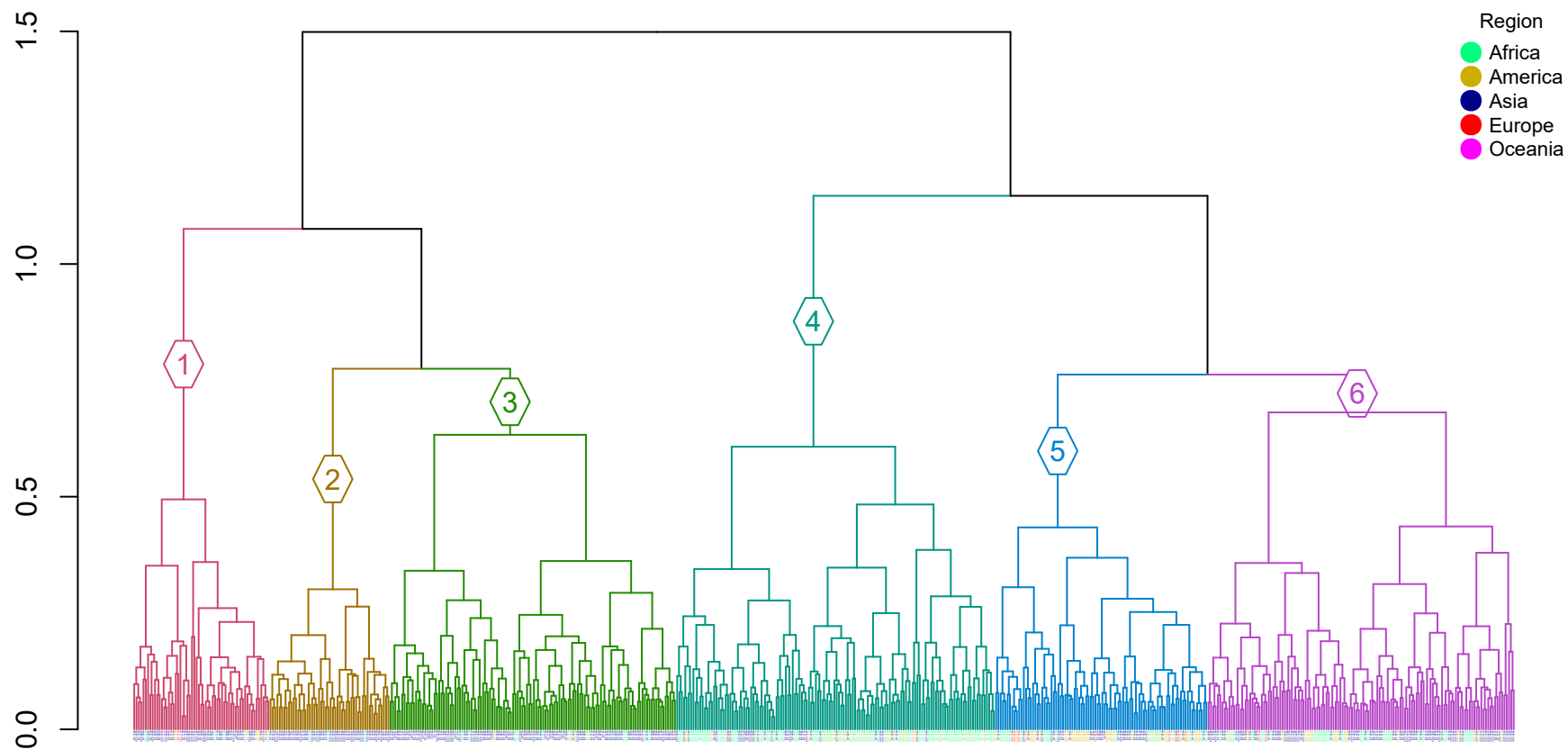

Supplement: Supplementary Figure 1 — Dendrogram constructed based on Gower’s distance using Ward. D2 clustering algorithm for 598 pigeonpea accessions. [file Image_1.pdf]
