# Supplementary material for: Grain Nutrients Variability in Pigeonpea Genebank Collection and Its Potential for Promoting Nutritional Security in Dryland Ecologies
Source: Front Plant Sci. 2022 Jul 11;13:934296. doi: 10.3389/fpls.2022.934296 (PMC9310011; doi:10.3389/fpls.2022.934296)
Supplement: Supplementary Table 1 — Metadata of the 600 pigeonpea accessions and four checks evaluated for agronomic traits and grain nutrients during the 2019 and 2020 rainy seasons at ICRISAT, India. [file Data_Sheet_1.docx]

**Grain Nutrients Variability in Pigeonpea Genebank Collection and its Potential for Promoting Nutritional Security in Dryland Ecologies**

**Supplementary Table 1.** Metadata of 600 pigeonpea accessions and four checks evaluated for agronomic traits and grain nutrients during 2019 and 2020 rainy seasons at ICRISAT, India

| **S. No** | **Accession** | **Alternate ID** | **Biological status** | **Country of collection** | **Region** | **DOI** |
| --- | --- | --- | --- | --- | --- | --- |
| 1 | ICP 78 | P 1883; PI 395330 | Landrace | India | Asia | https://doi.org/10.18730/PXKM9 |
| 2 | ICP 81 | P 1943 | Landrace | India | Asia | https://doi.org/10.18730/PXKPB |
| 3 | ICP 230 | P 2366; PI 395712 | Landrace | India | Asia | https://doi.org/10.18730/PXPDR |
| 4 | ICP 232 | P 2384; PI 395725 | Landrace | India | Asia | https://doi.org/10.18730/PXPFT |
| 5 | ICP 248 | P 2443; PI 395777 | Landrace | India | Asia | https://doi.org/10.18730/PXPR= |
| 6 | ICP 275 | P 2577/1 | Landrace | India | Asia | https://doi.org/10.18730/PXQ5B |
| 7 | ICP 312 | P 2908; PI 396174 | Landrace | India | Asia | https://doi.org/10.18730/PXQQX |
| 8 | ICP 325 | P 2993; PI 396246 | Landrace | India | Asia | <https://doi.org/10.18730/PXQX=> |
| 9 | ICP 474 | P 4498; No. 20-36 | Breeding line | India | Asia | https://doi.org/10.18730/PXTB2 |
| 10 | ICP 656 | P 612; PI 394467 | Landrace | India | Asia | https://doi.org/10.18730/PXX2F |
| 11 | ICP 769 | P 2088; PI 395494 | Landrace | India | Asia | https://doi.org/10.18730/PXYHS |
| 12 | ICP 774 | P 2279; PI 395643 | Landrace | India | Asia | https://doi.org/10.18730/PXYKV |
| 13 | ICP 841 | P 2725; PI 396026 | Landrace | India | Asia | https://doi.org/10.18730/PY020 |
| 14 | ICP 844 | P 2740/1 | Landrace | India | Asia | https://doi.org/10.18730/PY053 |
| 15 | ICP 845 | P 2744; PI 396042 | Landrace | India | Asia | https://doi.org/10.18730/PY064 |
| 16 | ICP 846 | P 2744-1 | Landrace | India | Asia | https://doi.org/10.18730/PY075 |
| 17 | ICP 848 | P 2746; PI 396043 | Landrace | India | Asia | https://doi.org/10.18730/PY086 |
| 18 | ICP 854 | P 2754-1 | Landrace | India | Asia | https://doi.org/10.18730/PY0DB |
| 19 | ICP 877 | P 2824; PI 396111 | Landrace | India | Asia | https://doi.org/10.18730/PY0YW |
| 20 | ICP 987 | P 3616; PI 396769 | Landrace | India | Asia | https://doi.org/10.18730/PY2SD |
| 21 | ICP 1000 | P 4123; MS 8906 | Breeding line | India | Asia | https://doi.org/10.18730/PY35S |
| 22 | ICP 1047 | P 4300; PI 397358 | Landrace | India | Asia | https://doi.org/10.18730/PY4AS |
| 23 | ICP 1050 | P 4361; PI 397396 | Landrace | India | Asia | https://doi.org/10.18730/PY4CV |
| 24 | ICP 1143 | P 604-120-2 | Landrace | India | Asia | https://doi.org/10.18730/PY6MS |
| 25 | ICP 1189 | P 85; PI 394079 | Landrace | India | Asia | https://doi.org/10.18730/PY7WW |
| 26 | ICP 1224 | P 1651; PI 395122 | Landrace | India | Asia | https://doi.org/10.18730/PY8TN |
| 27 | ICP 1261 | P 1818; PI 395270 | Landrace | India | Asia | https://doi.org/10.18730/PY9E4 |
| 28 | ICP 1458 | P 1918; PI 395357 | Landrace | India | Asia | https://doi.org/10.18730/PYBV7 |
| 29 | ICP 1514 | P 2098-2 | Landrace | India | Asia | https://doi.org/10.18730/PYCFV |
| 30 | ICP 1523 | P 2399; PI 395740 | Landrace | India | Asia | https://doi.org/10.18730/PYCJY |
| 31 | ICP 1529 | P 3659; PI 396807 | Landrace | India | Asia | https://doi.org/10.18730/PYCN~ |
| 32 | ICP 1644 | P 15-107-2-1 | Landrace | India | Asia | https://doi.org/10.18730/PYEJM |
| 33 | ICP 1739 | P 1648; PI 395119 | Landrace | India | Asia | https://doi.org/10.18730/PYFYV |
| 34 | ICP 1758 | P 1704/1-1 | Landrace | India | Asia | https://doi.org/10.18730/PYG6= |
| 35 | ICP 1889 | P 2353-7 | Landrace | India | Asia | https://doi.org/10.18730/PYJ8V |
| 36 | ICP 2068 | P 1657/1-1 | Landrace | India | Asia | https://doi.org/10.18730/PYMVU |
| 37 | ICP 2133 | P 1806/1 | Landrace | India | Asia | https://doi.org/10.18730/PYNNS |
| 38 | ICP 2155 | P 1870; PI 395318 | Landrace | India | Asia | https://doi.org/10.18730/PYNW* |
| 39 | ICP 2309 | P 3685 | Landrace | Nepal | Asia | https://doi.org/10.18730/PYQPG |
| 40 | ICP 2630 | P 2828 | Landrace | India | Asia | https://doi.org/10.18730/PYWPW |
| 41 | ICP 2860 | P 1678; PI 395147 | Landrace | India | Asia | https://doi.org/10.18730/PZ0RA |
| 42 | ICP 2889 | P 1793/1 | Landrace | India | Asia | https://doi.org/10.18730/PZ17S |
| 43 | ICP 3135 | P 2774; PI 396067 | Landrace | India | Asia | https://doi.org/10.18730/PZ4X* |
| 44 | ICP 3451 | P 647; PI 394482 | Landrace | India | Asia | https://doi.org/10.18730/PZA7H |
| 45 | ICP 3516 | P 1798; PI 395250 | Landrace | India | Asia | https://doi.org/10.18730/PZB49 |
| 46 | ICP 3808 | P 986; No 148 | Improved cultivar | India | Asia | https://doi.org/10.18730/PZGM0 |
| 47 | ICP 3852 | P 2916 | Landrace | India | Asia | https://doi.org/10.18730/PZHFV |
| 48 | ICP 3878 | P 4973; Banjaria | Landrace | India | Asia | https://doi.org/10.18730/PZJ07 |
| 49 | ICP 3904 | P 171; PI 394142 | Landrace | India | Asia | https://doi.org/10.18730/PZJBJ |
| 50 | ICP 3908 | P 175; PI 394146 | Landrace | India | Asia | https://doi.org/10.18730/PZJEN |
| 51 | ICP 3962 | P 4336; PI 397378 | Landrace | India | Asia | https://doi.org/10.18730/PZK79 |
| 52 | ICP 4017 | P 991-2; Jalgaon 1-1 | Landrace | India | Asia | https://doi.org/10.18730/PZKVX |
| 53 | ICP 4370 | P 144; PI 394123 | Landrace | India | Asia | https://doi.org/10.18730/PZRN= |
| 54 | ICP 4379 | P 282; PI 394462 | Landrace | India | Asia | https://doi.org/10.18730/PZRT3 |
| 55 | ICP 4386 | P 629; PI 394474 | Landrace | India | Asia | https://doi.org/10.18730/PZRZ8 |
| 56 | ICP 4398 | P 1165; BG 1 | Breeding line | Guyana | America | https://doi.org/10.18730/PZS6F |
| 57 | ICP 4400 | P 1167; BG 5 | Breeding line | Guyana | America | https://doi.org/10.18730/PZS7G |
| 58 | ICP 4628 | P 41-2-2 | Landrace | India | Asia | https://doi.org/10.18730/PZWD7 |
| 59 | ICP 4701 | P 32-143-1-1 | Landrace | India | Asia | https://doi.org/10.18730/PZXE3 |
| 60 | ICP 4729 | P 3251 | Landrace | India | Asia | https://doi.org/10.18730/PZXWH |
| 61 | ICP 4928 | P 923; HY 9 | Improved cultivar | India | Asia | https://doi.org/10.18730/Q0106 |
| 62 | ICP 4929 | P 923-1; HY 9 | Improved cultivar | India | Asia | https://doi.org/10.18730/Q0117 |
| 63 | ICP 5175 | P 1066/1-1; S BR 10 | Breeding line | India | Asia | https://doi.org/10.18730/Q04E5 |
| 64 | ICP 5322 | P 3152; PI 396353 | Landrace | India | Asia | https://doi.org/10.18730/Q074H |
| 65 | ICP 5325 | P 3155/1 | Landrace | India | Asia | https://doi.org/10.18730/Q076K |
| 66 | ICP 5335 | P 3173; PI 396404 | Landrace | India | Asia | https://doi.org/10.18730/Q079P |
| 67 | ICP 5344 | P 3300 | Landrace | India | Asia | https://doi.org/10.18730/Q07FW |
| 68 | ICP 5353 | P 3325; PI 396523 | Landrace | India | Asia | https://doi.org/10.18730/Q07N$ |
| 69 | ICP 5369 | P 3425; PI 396610 | Landrace | India | Asia | https://doi.org/10.18730/Q07X5 |
| 70 | ICP 5372 | P 3435; PI 396617 | Landrace | India | Asia | https://doi.org/10.18730/Q07Z7 |
| 71 | ICP 5375 | P 3436; PI 396618 | Landrace | India | Asia | https://doi.org/10.18730/Q0808 |
| 72 | ICP 5426 | P 3567; PI 396730 | Landrace | India | Asia | https://doi.org/10.18730/Q08PY |
| 73 | ICP 5428 | P 3568; PI 396731 | Landrace | India | Asia | https://doi.org/10.18730/Q08QZ |
| 74 | ICP 5449 | P 4479; HY 4 | Improved cultivar | India | Asia | https://doi.org/10.18730/Q0914 |
| 75 | ICP 5493 | P 4664; EB 3 | Breeding line | India | Asia | https://doi.org/10.18730/Q0A20 |
| 76 | ICP 5543 | P 145-3 | Landrace | India | Asia | https://doi.org/10.18730/Q0B70 |
| 77 | ICP 5545 | P 145-1 | Landrace | India | Asia | https://doi.org/10.18730/Q0B92 |
| 78 | ICP 5546 | P 145-1-1 | Landrace | India | Asia | https://doi.org/10.18730/Q0BA3 |
| 79 | ICP 5564 | P 59; PI 394054 | Landrace | India | Asia | https://doi.org/10.18730/Q0BPF |
| 80 | ICP 5632 | P 788; PI 394583 | Landrace | India | Asia | https://doi.org/10.18730/Q0CM8 |
| 81 | ICP 5664 | P 3262; PI 396473 | Landrace | India | Asia | https://doi.org/10.18730/Q0D4R |
| 82 | ICP 5668 | P 3266; PI 396476 | Landrace | India | Asia | https://doi.org/10.18730/Q0D6T |
| 83 | ICP 5673 | P 3358; PI 396551 | Landrace | India | Asia | https://doi.org/10.18730/Q0D7V |
| 84 | ICP 5694 | P 207; PI 394172 | Landrace | India | Asia | https://doi.org/10.18730/Q0DF= |
| 85 | ICP 5695 | P 260/2 | Landrace | India | Asia | https://doi.org/10.18730/Q0DGU |
| 86 | ICP 5779 | P 17-2 | Landrace | India | Asia | https://doi.org/10.18730/Q0ET4 |
| 87 | ICP 5795 | P 138; PI 394117 | Landrace | India | Asia | https://doi.org/10.18730/Q0F3D |
| 88 | ICP 5825 | P 685; PI 394513 | Landrace | India | Asia | https://doi.org/10.18730/Q0FGT |
| 89 | ICP 5836 | P 793; PI 394527 | Landrace | India | Asia | https://doi.org/10.18730/Q0FNZ |
| 90 | ICP 5860 | P 3013 | Landrace | India | Asia | https://doi.org/10.18730/Q0G05 |
| 91 | ICP 5893 | P 3189; PI 396417 | Landrace | India | Asia | https://doi.org/10.18730/Q0GHP |
| 92 | ICP 5914 | P 3327; PI 396525 | Landrace | India | Asia | https://doi.org/10.18730/Q0GW~ |
| 93 | ICP 5915 | P 3343; PI 396538 | Landrace | India | Asia | https://doi.org/10.18730/Q0GX$ |
| 94 | ICP 5917 | P 3356; PI 396549 | Landrace | India | Asia | https://doi.org/10.18730/Q0GZU |
| 95 | ICP 5923 | P 3462; PI 396639 | Landrace | India | Asia | https://doi.org/10.18730/Q0H22 |
| 96 | ICP 5925 | P 3492; PI 396668 | Landrace | India | Asia | https://doi.org/10.18730/Q0H33 |
| 97 | ICP 5928 | P 3496; PI 396672 | Landrace | India | Asia | https://doi.org/10.18730/Q0H44 |
| 98 | ICP 5950 | P 3589; PI 396747 | Landrace | India | Asia | https://doi.org/10.18730/Q0HDD |
| 99 | ICP 5960 | P 3600 | Landrace | India | Asia | https://doi.org/10.18730/Q0HHH |
| 100 | ICP 5967 | P 3629; PI 396781 | Landrace | India | Asia | https://doi.org/10.18730/Q0HMM |
| 101 | ICP 5969 | P 3636; PI 396787 | Landrace | India | Asia | https://doi.org/10.18730/Q0HNN |
| 102 | ICP 5970 | P 3636-1 | Landrace | India | Asia | https://doi.org/10.18730/Q0HPP |
| 103 | ICP 5991 | P 4333 | Landrace | India | Asia | https://doi.org/10.18730/Q0J1~ |
| 104 | ICP 5995 | P 4349; PI 397388 | Landrace | India | Asia | https://doi.org/10.18730/Q0J3= |
| 105 | ICP 5999 | P 4581; PLA 191 | Landrace | India | Asia | https://doi.org/10.18730/Q0J61 |
| 106 | ICP 6027 | P 27; PI 394024 | Landrace | India | Asia | https://doi.org/10.18730/Q0JMF |
| 107 | ICP 6056 | P 402; PI 394319 | Landrace | India | Asia | https://doi.org/10.18730/Q0K5* |
| 108 | ICP 6081 | P 677; PI 394507 | Landrace | India | Asia | https://doi.org/10.18730/Q0KH7 |
| 109 | ICP 6163 | P 2481; PI 395812 | Landrace | India | Asia | https://doi.org/10.18730/Q0MZG |
| 110 | ICP 6165 | P 2484; PI 395814 | Landrace | India | Asia | https://doi.org/10.18730/Q0N0H |
| 111 | ICP 6219 | P 3116 | Landrace | India | Asia | https://doi.org/10.18730/Q0P0C |
| 112 | ICP 6229 | P 3136; PI 396369 | Landrace | India | Asia | https://doi.org/10.18730/Q0P5H |
| 113 | ICP 6233 | P 3139-1 | Landrace | India | Asia | https://doi.org/10.18730/Q0P7K |
| 114 | ICP 6241 | P 3209; PI 396432 | Landrace | India | Asia | https://doi.org/10.18730/Q0PBQ |
| 115 | ICP 6249 | P 3229/2 | Landrace | India | Asia | https://doi.org/10.18730/Q0PET |
| 116 | ICP 6272 | P 3400 | Landrace | India | Asia | https://doi.org/10.18730/Q0PV2 |
| 117 | ICP 6288 | P 3486; PI 396662 | Landrace | India | Asia | https://doi.org/10.18730/Q0Q3A |
| 118 | ICP 6304 | P 4481; Hybrid 2 | Improved cultivar | India | Asia | https://doi.org/10.18730/Q0QGQ |
| 119 | ICP 6386 | P 5120/2; TUR 127 | Landrace | India | Asia | https://doi.org/10.18730/Q0RY* |
| 120 | ICP 6399 | EC 100465 | Landrace | Peru | America | https://doi.org/10.18730/Q0S41 |
| 121 | ICP 6400 | EC 100465-1 | Landrace | Peru | America | https://doi.org/10.18730/Q0S52 |
| 122 | ICP 6410 | P 584-1 | Landrace | India | Asia | https://doi.org/10.18730/Q0SA7 |
| 123 | ICP 6427 | P 427-1 | Landrace | India | Asia | https://doi.org/10.18730/Q0SNJ |
| 124 | ICP 6486 | P 2142; PI 395543 | Landrace | India | Asia | https://doi.org/10.18730/Q0TXN |
| 125 | ICP 6487 | P 2143; PI 395544 | Landrace | India | Asia | https://doi.org/10.18730/Q0TYP |
| 126 | ICP 6560 | P 2441; PI 395775 | Landrace | India | Asia | https://doi.org/10.18730/Q0X1F |
| 127 | ICP 6632 | P 2680/1 | Landrace | India | Asia | https://doi.org/10.18730/Q0Z9D |
| 128 | ICP 6659 | P 2721; PI 396023 | Landrace | India | Asia | https://doi.org/10.18730/Q1043 |
| 129 | ICP 6669 | P 2735; PI 396033 | Landrace | India | Asia | https://doi.org/10.18730/Q10ED |
| 130 | ICP 6672 | P 2742/1 | Landrace | India | Asia | https://doi.org/10.18730/Q10HG |
| 131 | ICP 6700 | P 2809; PI 396097 | Landrace | India | Asia | https://doi.org/10.18730/Q11C6 |
| 132 | ICP 6705 | P 2823; PI 396110 | Landrace | India | Asia | https://doi.org/10.18730/Q11HB |
| 133 | ICP 6713 | P 2836; PI 396121 | Landrace | India | Asia | https://doi.org/10.18730/Q11SK |
| 134 | ICP 6720 | P 2857; PI 396137 | Landrace | India | Asia | https://doi.org/10.18730/Q120T |
| 135 | ICP 6722 | P 2859; PI 396139 | Landrace | India | Asia | https://doi.org/10.18730/Q122W |
| 136 | ICP 6735 | P 2933 | Landrace | India | Asia | https://doi.org/10.18730/Q12F4 |
| 137 | ICP 6743 | P 2973; PI 396228 | Landrace | India | Asia | https://doi.org/10.18730/Q12QC |
| 138 | ICP 6750 | P 3011; PI 396260 | Landrace | India | Asia | https://doi.org/10.18730/Q12YK |
| 139 | ICP 6758 | P 3057; PI 396296 | Landrace | India | Asia | https://doi.org/10.18730/Q135T |
| 140 | ICP 6789 | P 3143; PI 396375 | Landrace | India | Asia | https://doi.org/10.18730/Q144M |
| 141 | ICP 6814 | P 3211 | Landrace | India | Asia | https://doi.org/10.18730/Q14X8 |
| 142 | ICP 6834 | P 3321; PI 396519 | Landrace | India | Asia | https://doi.org/10.18730/Q15HW |
| 143 | ICP 6864 | P 3436; PI 396618 | Landrace | India | Asia | https://doi.org/10.18730/Q16FN |
| 144 | ICP 6882 | P 3526; PI 396699 | Landrace | India | Asia | https://doi.org/10.18730/Q1712 |
| 145 | ICP 6914 | Code No. 2 | Breeding line | Trinidad and Tobago | America | https://doi.org/10.18730/Q180~ |
| 146 | ICP 6915 | Code No. 3 | Breeding line | Trinidad and Tobago | America | https://doi.org/10.18730/Q181$ |
| 147 | ICP 6964 | Local variety | Breeding line | Thailand | Asia | https://doi.org/10.18730/Q19J9 |
| 148 | ICP 7024 | - | Landrace | India | Asia | https://doi.org/10.18730/Q1BE* |
| 149 | ICP 7028 | - | Landrace | India | Asia | https://doi.org/10.18730/Q1BJU |
| 150 | ICP 7035 | - | Landrace | India | Asia | https://doi.org/10.18730/Q1BS6 |
| 151 | ICP 7040 | - | Landrace | India | Asia | https://doi.org/10.18730/Q1BYB |
| 152 | ICP 7041 | - | Landrace | India | Asia | https://doi.org/10.18730/Q1BZC |
| 153 | ICP 7044 | - | Landrace | India | Asia | https://doi.org/10.18730/Q1C2F |
| 154 | ICP 7057 | - | Landrace | India | Asia | https://doi.org/10.18730/Q1CFW |
| 155 | ICP 7081 | - | Landrace | India | Asia | https://doi.org/10.18730/Q1D7F |
| 156 | ICP 7109 | - | Landrace | India | Asia | https://doi.org/10.18730/Q1E36 |
| 157 | ICP 7139 | EC 109888; Sel. XIX | Breeding line | Sri Lanka | Asia | https://doi.org/10.18730/Q1F1U |
| 158 | ICP 7336 | - | Landrace | India | Asia | https://doi.org/10.18730/Q1N6B |
| 159 | ICP 7337 | - | Landrace | India | Asia | https://doi.org/10.18730/Q1N7C |
| 160 | ICP 7344 | - | Landrace | India | Asia | https://doi.org/10.18730/Q1NEK |
| 161 | ICP 7347 | - | Landrace | India | Asia | https://doi.org/10.18730/Q1NHP |
| 162 | ICP 7399 | - | Landrace | India | Asia | https://doi.org/10.18730/Q1Q50 |
| 163 | ICP 7402 | - | Landrace | India | Asia | https://doi.org/10.18730/Q1Q83 |
| 164 | ICP 7407 | - | Landrace | India | Asia | https://doi.org/10.18730/Q1QD8 |
| 165 | ICP 7412 | - | Landrace | India | Asia | https://doi.org/10.18730/Q1QJD |
| 166 | ICP 7422 | - | Landrace | India | Asia | https://doi.org/10.18730/Q1QWQ |
| 167 | ICP 7439 | - | Landrace | India | Asia | https://doi.org/10.18730/Q1RD3 |
| 168 | ICP 7443 | - | Landrace | India | Asia | https://doi.org/10.18730/Q1RH7 |
| 169 | ICP 7459 | - | Landrace | India | Asia | https://doi.org/10.18730/Q1S1Q |
| 170 | ICP 7527 | - | Landrace | India | Asia | https://doi.org/10.18730/Q1TX9 |
| 171 | ICP 7531 | - | Landrace | India | Asia | https://doi.org/10.18730/Q1V1D |
| 172 | ICP 7533 | - | Landrace | India | Asia | https://doi.org/10.18730/Q1V3F |
| 173 | ICP 7563 | - | Landrace | India | Asia | https://doi.org/10.18730/Q1W18 |
| 174 | ICP 7568 | - | Landrace | India | Asia | https://doi.org/10.18730/Q1W6D |
| 175 | ICP 7578 | - | Landrace | India | Asia | https://doi.org/10.18730/Q1WGQ |
| 176 | ICP 7588 | - | Landrace | India | Asia | https://doi.org/10.18730/Q1WT~ |
| 177 | ICP 7594 | - | Landrace | India | Asia | https://doi.org/10.18730/Q1X02 |
| 178 | ICP 7595 | - | Landrace | India | Asia | https://doi.org/10.18730/Q1X13 |
| 179 | ICP 7608 | CO 1 | Landrace | India | Asia | https://doi.org/10.18730/Q1XEG |
| 180 | ICP 7621 | Brazil 5 | Landrace | Colombia | America | https://doi.org/10.18730/Q1XVX |
| 181 | ICP 7650 | 4S 2209 | Landrace | Nigeria | Africa | https://doi.org/10.18730/Q1YRN |
| 182 | ICP 7652 | 4S 2220 | Landrace | Nigeria | Africa | https://doi.org/10.18730/Q1YTQ |
| 183 | ICP 7653 | 4S 2263 | Landrace | Nigeria | Africa | https://doi.org/10.18730/Q1YVR |
| 184 | ICP 7804 | Anni 1-2-1-7 | Landrace | India | Asia | https://doi.org/10.18730/Q23JV |
| 185 | ICP 7866 | - | Landrace | India | Asia | https://doi.org/10.18730/Q25GF |
| 186 | ICP 7867 | - | Landrace | India | Asia | https://doi.org/10.18730/Q25HG |
| 187 | ICP 7869 | - | Landrace | India | Asia | https://doi.org/10.18730/Q25KJ |
| 188 | ICP 7870 | - | Landrace | India | Asia | https://doi.org/10.18730/Q25MK |
| 189 | ICP 7903 | - | Landrace | India | Asia | https://doi.org/10.18730/Q26NF |
| 190 | ICP 7942 | - | Landrace | India | Asia | https://doi.org/10.18730/Q27WH |
| 191 | ICP 7974 | - | Landrace | India | Asia | https://doi.org/10.18730/Q28WC |
| 192 | ICP 7982 | - | Landrace | India | Asia | https://doi.org/10.18730/Q294M |
| 193 | ICP 7995 | - | Landrace | India | Asia | https://doi.org/10.18730/Q29H~ |
| 194 | ICP 8008 | - | Landrace | India | Asia | https://doi.org/10.18730/Q29Y9 |
| 195 | ICP 8015 | - | Landrace | India | Asia | https://doi.org/10.18730/Q2A5G |
| 196 | ICP 8019 | - | Landrace | India | Asia | https://doi.org/10.18730/Q2A9M |
| 197 | ICP 8034 | - | Landrace | India | Asia | https://doi.org/10.18730/Q2AR= |
| 198 | ICP 8035 | - | Landrace | India | Asia | https://doi.org/10.18730/Q2ASU |
| 199 | ICP 8042 | - | Landrace | India | Asia | https://doi.org/10.18730/Q2B06 |
| 200 | ICP 8045 | - | Landrace | India | Asia | https://doi.org/10.18730/Q2B39 |
| 201 | ICP 8098 | - | Landrace | India | Asia | https://doi.org/10.18730/Q2CRS |
| 202 | ICP 8099 | - | Landrace | India | Asia | https://doi.org/10.18730/Q2CST |
| 203 | ICP 8101 | - | Landrace | India | Asia | https://doi.org/10.18730/Q2CVW |
| 204 | ICP 8102 | - | Landrace | India | Asia | https://doi.org/10.18730/Q2CWX |
| 205 | ICP 8107 | - | Landrace | India | Asia | https://doi.org/10.18730/Q2D1$ |
| 206 | ICP 8117 | - | Landrace | India | Asia | https://doi.org/10.18730/Q2DB7 |
| 207 | ICP 8118 | - | Landrace | India | Asia | https://doi.org/10.18730/Q2DC8 |
| 208 | ICP 8119 | - | Landrace | India | Asia | https://doi.org/10.18730/Q2DD9 |
| 209 | ICP 8132 | - | Landrace | India | Asia | https://doi.org/10.18730/Q2DTP |
| 210 | ICP 8165 | - | Landrace | India | Asia | https://doi.org/10.18730/Q2EVJ |
| 211 | ICP 8166 | - | Landrace | India | Asia | https://doi.org/10.18730/Q2EWK |
| 212 | ICP 8177 | Dupuy 3 | Landrace | Guadeloupe | America | https://doi.org/10.18730/Q2F7Y |
| 213 | ICP 8178 | - | Landrace | Guadeloupe | America | https://doi.org/10.18730/Q2F8Z |
| 214 | ICP 8193 | 63/147 | Landrace | Senegal | Africa | https://doi.org/10.18730/Q2FQ9 |
| 215 | ICP 8194 | 68/182 | Landrace | Senegal | Africa | https://doi.org/10.18730/Q2FRA |
| 216 | ICP 8202 | 68/196 | Landrace | Senegal | Africa | https://doi.org/10.18730/Q2G0J |
| 217 | ICP 8353 | - | Landrace | India | Asia | https://doi.org/10.18730/Q2MQN |
| 218 | ICP 8354 | - | Landrace | India | Asia | https://doi.org/10.18730/Q2MRP |
| 219 | ICP 8355 | - | Landrace | India | Asia | https://doi.org/10.18730/Q2MSQ |
| 220 | ICP 8356 | - | Landrace | India | Asia | https://doi.org/10.18730/Q2MTR |
| 221 | ICP 8392 | - | Landrace | India | Asia | https://doi.org/10.18730/Q2NYQ |
| 222 | ICP 8396 | - | Landrace | India | Asia | https://doi.org/10.18730/Q2P2V |
| 223 | ICP 8406 | - | Landrace | India | Asia | https://doi.org/10.18730/Q2PC0 |
| 224 | ICP 8407 | - | Landrace | India | Asia | https://doi.org/10.18730/Q2PD1 |
| 225 | ICP 8408 | - | Landrace | India | Asia | https://doi.org/10.18730/Q2PE2 |
| 226 | ICP 8412 | - | Landrace | India | Asia | https://doi.org/10.18730/Q2PJ6 |
| 227 | ICP 8424 | Dhar 8 | Landrace | India | Asia | https://doi.org/10.18730/Q2PYJ |
| 228 | ICP 8454 | P 450 | Landrace | India | Asia | https://doi.org/10.18730/Q2QWB |
| 229 | ICP 8457 | P 476 | Landrace | India | Asia | https://doi.org/10.18730/Q2QZE |
| 230 | ICP 8519 | - | Landrace | India | Asia | https://doi.org/10.18730/Q2SX2 |
| 231 | ICP 8533 | - | Landrace | India | Asia | https://doi.org/10.18730/Q2TBG |
| 232 | ICP 8534 | - | Landrace | India | Asia | https://doi.org/10.18730/Q2TCH |
| 233 | ICP 8588 | - | Landrace | India | Asia | https://doi.org/10.18730/Q2W2$ |
| 234 | ICP 8596 | - | Landrace | India | Asia | https://doi.org/10.18730/Q2WA5 |
| 235 | ICP 8628 | - | Landrace | India | Asia | https://doi.org/10.18730/Q2XA0 |
| 236 | ICP 8636 | - | Landrace | India | Asia | https://doi.org/10.18730/Q2XJ8 |
| 237 | ICP 8648 | - | Landrace | India | Asia | https://doi.org/10.18730/Q2XYM |
| 238 | ICP 8670 | - | Landrace | India | Asia | https://doi.org/10.18730/Q2YM5 |
| 239 | ICP 8675 | - | Landrace | India | Asia | https://doi.org/10.18730/Q2YSA |
| 240 | ICP 8741 | Ajmer 4 | Landrace | India | Asia | https://doi.org/10.18730/Q30V2 |
| 241 | ICP 8807 | Hissar 8 | Landrace | India | Asia | https://doi.org/10.18730/Q32XZ |
| 242 | ICP 8820 | Kuselghat 4 | Landrace | India | Asia | https://doi.org/10.18730/Q33A7 |
| 243 | ICP 8877 | - | Landrace | India | Asia | https://doi.org/10.18730/Q353V |
| 244 | ICP 9035 | - | Landrace | India | Asia | https://doi.org/10.18730/Q3A10 |
| 245 | ICP 9037 | - | Landrace | India | Asia | https://doi.org/10.18730/Q3A32 |
| 246 | ICP 9038 | - | Landrace | India | Asia | https://doi.org/10.18730/Q3A43 |
| 247 | ICP 9040 | - | Landrace | India | Asia | https://doi.org/10.18730/Q3A65 |
| 248 | ICP 9049 | - | Landrace | India | Asia | https://doi.org/10.18730/Q3AFE |
| 249 | ICP 9055 | - | Landrace | India | Asia | https://doi.org/10.18730/Q3ANM |
| 250 | ICP 9122 | WIR 3 (ATG) | Landrace | Russian Federation | Europe | https://doi.org/10.18730/Q3CRD |
| 251 | ICP 9123 | WIR 274 (IND) | Landrace | Russian Federation | Europe | https://doi.org/10.18730/Q3CSE |
| 252 | ICP 9124 | SCI-F2 | Landrace | Venezuela | America | https://doi.org/10.18730/Q3CTF |
| 253 | ICP 9131 | - | Landrace | Kenya | Africa | https://doi.org/10.18730/Q3D1P |
| 254 | ICP 9132 | - | Landrace | Kenya | Africa | https://doi.org/10.18730/Q3D2Q |
| 255 | ICP 9137 | - | Landrace | Kenya | Africa | https://doi.org/10.18730/Q3D7W |
| 256 | ICP 9139 | - | Landrace | Kenya | Africa | https://doi.org/10.18730/Q3D9Y |
| 257 | ICP 9141 | - | Landrace | Kenya | Africa | https://doi.org/10.18730/Q3DB* |
| 258 | ICP 9144 | - | Landrace | Kenya | Africa | https://doi.org/10.18730/Q3DE= |
| 259 | ICP 9146 | - | Landrace | Kenya | Africa | https://doi.org/10.18730/Q3DG0 |
| 260 | ICP 9147 | - | Landrace | Kenya | Africa | https://doi.org/10.18730/Q3DH1 |
| 261 | ICP 9150 | - | Landrace | Kenya | Africa | https://doi.org/10.18730/Q3DM4 |
| 262 | ICP 9152 | - | Landrace | Kenya | Africa | https://doi.org/10.18730/Q3DP6 |
| 263 | ICP 9166 | - | Landrace | Kenya | Africa | https://doi.org/10.18730/Q3E4M |
| 264 | ICP 9185 | - | Landrace | Kenya | Africa | https://doi.org/10.18730/Q3EQ2 |
| 265 | ICP 9190 | - | Landrace | Kenya | Africa | https://doi.org/10.18730/Q3EW7 |
| 266 | ICP 9192 | - | Landrace | Kenya | Africa | https://doi.org/10.18730/Q3EY9 |
| 267 | ICP 9317 | PI 395153; P 1684 | Landrace | India | Asia | https://doi.org/10.18730/Q3JVQ |
| 268 | ICP 9361 | PI 395244; P 1791 | Landrace | India | Asia | https://doi.org/10.18730/Q3M7Y |
| 269 | ICP 9444 | PI 396022; P 2719 | Landrace | India | Asia | https://doi.org/10.18730/Q3PT2 |
| 270 | ICP 9542 | PI 396732; P 3569 | Landrace | India | Asia | https://doi.org/10.18730/Q3SWT |
| 271 | ICP 9877 | - | Landrace | Puerto Rico | America | https://doi.org/10.18730/Q44BW |
| 272 | ICP 10086 | - | Landrace | India | Asia | https://doi.org/10.18730/Q4AWF |
| 273 | ICP 10088 | - | Landrace | India | Asia | https://doi.org/10.18730/Q4AYH |
| 274 | ICP 10102 | - | Landrace | India | Asia | https://doi.org/10.18730/Q4BCZ |
| 275 | ICP 10113 | - | Landrace | India | Asia | https://doi.org/10.18730/Q4BQ5 |
| 276 | ICP 10118 | - | Landrace | India | Asia | https://doi.org/10.18730/Q4BWA |
| 277 | ICP 10176 | PI 394372; P 486 | Landrace | India | Asia | https://doi.org/10.18730/Q4DPZ |
| 278 | ICP 10298 | PI 395044; P 1565 | Landrace | India | Asia | https://doi.org/10.18730/Q4HG5 |
| 279 | ICP 10458 | PI 395753; P 2417 | Landrace | India | Asia | https://doi.org/10.18730/Q4PGH |
| 280 | ICP 10589 | PI 396444; P 3225 | Landrace | India | Asia | https://doi.org/10.18730/Q4TK0 |
| 281 | ICP 10600 | PI 396626; P 3447 | Landrace | India | Asia | https://doi.org/10.18730/Q4TYB |
| 282 | ICP 10618 | PI 396683; P 3510 | Landrace | India | Asia | https://doi.org/10.18730/Q4VGX |
| 283 | ICP 10876 | Dark brown | Landrace | China, | Asia | https://doi.org/10.18730/Q53JW |
| 284 | ICP 10880 | PI 275 | Landrace | Philippines | Asia | https://doi.org/10.18730/Q53P* |
| 285 | ICP 10889 | Collection No. 3 | Landrace | Indonesia | Asia | https://doi.org/10.18730/Q53Z4 |
| 286 | ICP 11348 | - | Landrace | Nepal | Asia | https://doi.org/10.18730/Q5JAK |
| 287 | ICP 11350 | - | Landrace | Nepal | Asia | https://doi.org/10.18730/Q5JCN |
| 288 | ICP 11362 | - | Landrace | Nepal | Asia | https://doi.org/10.18730/Q5JR~ |
| 289 | ICP 11369 | - | Landrace | Nepal | Asia | https://doi.org/10.18730/Q5JZ3 |
| 290 | ICP 11370 | - | Landrace | Nepal | Asia | https://doi.org/10.18730/Q5K04 |
| 291 | ICP 11387 | - | Landrace | Nepal | Asia | https://doi.org/10.18730/Q5KHN |
| 292 | ICP 11393 | - | Landrace | Nepal | Asia | https://doi.org/10.18730/Q5KQV |
| 293 | ICP 11396 | - | Landrace | Nepal | Asia | https://doi.org/10.18730/Q5KTY |
| 294 | ICP 11397 | - | Landrace | Nepal | Asia | https://doi.org/10.18730/Q5KVZ |
| 295 | ICP 11399 | - | Landrace | Nepal | Asia | https://doi.org/10.18730/Q5KX~ |
| 296 | ICP 11400 | - | Landrace | Nepal | Asia | https://doi.org/10.18730/Q5KY$ |
| 297 | ICP 11412 | - | Landrace | Bangladesh | Asia | https://doi.org/10.18730/Q5MA9 |
| 298 | ICP 11442 | - | Landrace | Malawi | Africa | https://doi.org/10.18730/Q5N82 |
| 299 | ICP 11446 | - | Landrace | Malawi | Africa | https://doi.org/10.18730/Q5NC6 |
| 300 | ICP 11464 | - | Landrace | Zambia | Africa | https://doi.org/10.18730/Q5NYR |
| 301 | ICP 11465 | - | Landrace | Zambia | Africa | https://doi.org/10.18730/Q5NZS |
| 302 | ICP 11466 | - | Landrace | Zambia | Africa | https://doi.org/10.18730/Q5P0T |
| 303 | ICP 11472 | - | Landrace | Sri Lanka | Asia | https://doi.org/10.18730/Q5P6* |
| 304 | ICP 11475 | - | Landrace | Sri Lanka | Asia | https://doi.org/10.18730/Q5P9= |
| 305 | ICP 11480 | FAO Acc 51-426; CITA 4 | Landrace | Nigeria | Africa | https://doi.org/10.18730/Q5PE3 |
| 306 | ICP 11483 | - | Landrace | Thailand | Asia | https://doi.org/10.18730/Q5PH6 |
| 307 | ICP 11485 | - | Landrace | Thailand | Asia | https://doi.org/10.18730/Q5PK8 |
| 308 | ICP 11486 | - | Landrace | Thailand | Asia | https://doi.org/10.18730/Q5PM9 |
| 309 | ICP 11487 | - | Landrace | Myanmar | Asia | https://doi.org/10.18730/Q5PNA |
| 310 | ICP 11491 | - | Landrace | Myanmar | Asia | https://doi.org/10.18730/Q5PSE |
| 311 | ICP 11811 | SCG 16-3 | Landrace | India | Asia | https://doi.org/10.18730/Q60S1 |
| 312 | ICP 11836 | SCG 22-1 | Landrace | India | Asia | https://doi.org/10.18730/Q61JT |
| 313 | ICP 11849 | SCG 25-3 | Landrace | India | Asia | https://doi.org/10.18730/Q61Z2 |
| 314 | ICP 11850 | SCG 25-4 | Landrace | India | Asia | https://doi.org/10.18730/Q6203 |
| 315 | ICP 11852 | SCG 25-6 | Landrace | India | Asia | https://doi.org/10.18730/Q6225 |
| 316 | ICP 11853 | SCG 26-1 | Landrace | India | Asia | https://doi.org/10.18730/Q6236 |
| 317 | ICP 11856 | SCG 27-1 | Landrace | India | Asia | https://doi.org/10.18730/Q6269 |
| 318 | ICP 11862 | SCG 29-1 | Landrace | India | Asia | https://doi.org/10.18730/Q62CF |
| 319 | ICP 11863 | SCG 29-2 | Landrace | India | Asia | https://doi.org/10.18730/Q62DG |
| 320 | ICP 11883 | SCG 38-2 | Landrace | India | Asia | https://doi.org/10.18730/Q631U |
| 321 | ICP 11980 | - | Landrace | Philippines | Asia | https://doi.org/10.18730/Q662P |
| 322 | ICP 11990 | - | Landrace | Philippines | Asia | https://doi.org/10.18730/Q66C* |
| 323 | ICP 11991 | - | Landrace | Philippines | Asia | https://doi.org/10.18730/Q66D~ |
| 324 | ICP 11995 | - | Landrace | Philippines | Asia | https://doi.org/10.18730/Q66H0 |
| 325 | ICP 12011 | - | Landrace | Tanzania | Africa | https://doi.org/10.18730/Q671G |
| 326 | ICP 12012 | - | Landrace | Tanzania | Africa | https://doi.org/10.18730/Q672H |
| 327 | ICP 12013 | - | Landrace | Tanzania | Africa | https://doi.org/10.18730/Q673J |
| 328 | ICP 12014 | - | Landrace | Tanzania | Africa | https://doi.org/10.18730/Q674K |
| 329 | ICP 12023 | - | Landrace | Tanzania | Africa | https://doi.org/10.18730/Q67DW |
| 330 | ICP 12024 | - | Landrace | Tanzania | Africa | https://doi.org/10.18730/Q67EX |
| 331 | ICP 12025 | - | Landrace | Tanzania | Africa | https://doi.org/10.18730/Q67FY |
| 332 | ICP 12026 | - | Landrace | Tanzania | Africa | https://doi.org/10.18730/Q67GZ |
| 333 | ICP 12027 | - | Landrace | Tanzania | Africa | https://doi.org/10.18730/Q67H* |
| 334 | ICP 12028 | - | Landrace | Tanzania | Africa | https://doi.org/10.18730/Q67J~ |
| 335 | ICP 12029 | - | Landrace | Tanzania | Africa | https://doi.org/10.18730/Q67K$ |
| 336 | ICP 12041 | - | Landrace | Tanzania | Africa | https://doi.org/10.18730/Q67Z9 |
| 337 | ICP 12042 | - | Landrace | Tanzania | Africa | https://doi.org/10.18730/Q680A |
| 338 | ICP 12043 | - | Landrace | Tanzania | Africa | https://doi.org/10.18730/Q681B |
| 339 | ICP 12047 | - | Landrace | Tanzania | Africa | https://doi.org/10.18730/Q685F |
| 340 | ICP 12048 | - | Landrace | Tanzania | Africa | https://doi.org/10.18730/Q686G |
| 341 | ICP 12049 | - | Landrace | Tanzania | Africa | https://doi.org/10.18730/Q687H |
| 342 | ICP 12050 | - | Landrace | Tanzania | Africa | https://doi.org/10.18730/Q688J |
| 343 | ICP 12051 | - | Landrace | Tanzania | Africa | https://doi.org/10.18730/Q689K |
| 344 | ICP 12062 | - | Landrace | Tanzania | Africa | https://doi.org/10.18730/Q68MY |
| 345 | ICP 12064 | - | Landrace | Tanzania | Africa | https://doi.org/10.18730/Q68P* |
| 346 | ICP 12065 | - | Landrace | Tanzania | Africa | https://doi.org/10.18730/Q68Q~ |
| 347 | ICP 12067 | - | Landrace | Tanzania | Africa | https://doi.org/10.18730/Q68S= |
| 348 | ICP 12068 | - | Landrace | Tanzania | Africa | https://doi.org/10.18730/Q68TU |
| 349 | ICP 12078 | - | Landrace | Tanzania | Africa | https://doi.org/10.18730/Q6949 |
| 350 | ICP 12095 | - | Landrace | Tanzania | Africa | https://doi.org/10.18730/Q69NT |
| 351 | ICP 12098 | - | Landrace | Tanzania | Africa | https://doi.org/10.18730/Q69RX |
| 352 | ICP 12129 | - | Landrace | Tanzania | Africa | https://doi.org/10.18730/Q6AQQ |
| 353 | ICP 12166 | - | Landrace | Thailand | Asia | https://doi.org/10.18730/Q6BWQ |
| 354 | ICP 12169 | - | Landrace | Thailand | Asia | https://doi.org/10.18730/Q6BZT |
| 355 | ICP 12172 | - | Landrace | Thailand | Asia | https://doi.org/10.18730/Q6C2X |
| 356 | ICP 12174 | - | Landrace | Thailand | Asia | https://doi.org/10.18730/Q6C4Z |
| 357 | ICP 12189 | JSK | Landrace | South Africa | Africa | https://doi.org/10.18730/Q6CK9 |
| 358 | ICP 12190 | - | Landrace | Ghana | Africa | https://doi.org/10.18730/Q6CMA |
| 359 | ICP 12261 | PI 394396; P 522 | Landrace | India | Asia | https://doi.org/10.18730/Q6EV7 |
| 360 | ICP 12264 | PI 394402; P 529 | Landrace | India | Asia | https://doi.org/10.18730/Q6EYA |
| 361 | ICP 12282 | PI 394508; P 678 | Landrace | India | Asia | https://doi.org/10.18730/Q6FGW |
| 362 | ICP 12465 | PI 396251; P 2999 | Landrace | India | Asia | https://doi.org/10.18730/Q6N7T |
| 363 | ICP 12506 | PI 396378; P 3147 | Landrace | India | Asia | https://doi.org/10.18730/Q6PGY |
| 364 | ICP 12538 | PI 396634; P 3455 | Landrace | India | Asia | https://doi.org/10.18730/Q6QGS |
| 365 | ICP 12551 | PI 396690; P 3517 | Landrace | India | Asia | https://doi.org/10.18730/Q6QX1 |
| 366 | ICP 12558 | PI 396716; P 3551 | Landrace | India | Asia | https://doi.org/10.18730/Q6R48 |
| 367 | ICP 12761 | - | Landrace | Philippines | Asia | https://doi.org/10.18730/Q6YFT |
| 368 | ICP 12763 | - | Landrace | Philippines | Asia | https://doi.org/10.18730/Q6YHW |
| 369 | ICP 12773 | - | Landrace | Philippines | Asia | https://doi.org/10.18730/Q6YV1 |
| 370 | ICP 12783 | - | Landrace | Tanzania | Africa | https://doi.org/10.18730/Q6Z5B |
| 371 | ICP 12788 | - | Landrace | Tanzania | Africa | https://doi.org/10.18730/Q6ZAG |
| 372 | ICP 12815 | - | Landrace | Tanzania | Africa | https://doi.org/10.18730/Q7056 |
| 373 | ICP 12816 | - | Landrace | Tanzania | Africa | https://doi.org/10.18730/Q7067 |
| 374 | ICP 12826 | - | Landrace | Tanzania | Africa | https://doi.org/10.18730/Q70GH |
| 375 | ICP 12828 | - | Landrace | Tanzania | Africa | https://doi.org/10.18730/Q70JK |
| 376 | ICP 12832 | Var. matiyambe | Landrace | Malawi | Africa | https://doi.org/10.18730/Q70PQ |
| 377 | ICP 12836 | - | Landrace | Mozambique | Africa | https://doi.org/10.18730/Q70TV |
| 378 | ICP 12837 | - | Landrace | Mozambique | Africa | https://doi.org/10.18730/Q70VW |
| 379 | ICP 12840 | - | Landrace | Mozambique | Africa | https://doi.org/10.18730/Q70YZ |
| 380 | ICP 12842 | - | Landrace | Mozambique | Africa | https://doi.org/10.18730/Q710~ |
| 381 | ICP 12843 | - | Landrace | Mozambique | Africa | https://doi.org/10.18730/Q711$ |
| 382 | ICP 12928 | - | Landrace | India | Asia | https://doi.org/10.18730/Q73P8 |
| 383 | ICP 12942 | - | Landrace | India | Asia | https://doi.org/10.18730/Q744P |
| 384 | ICP 13046 | - | Landrace | Kenya | Africa | https://doi.org/10.18730/Q77CF |
| 385 | ICP 13049 | - | Landrace | Kenya | Africa | https://doi.org/10.18730/Q77FJ |
| 386 | ICP 13105 | - | Landrace | Kenya | Africa | https://doi.org/10.18730/Q7970 |
| 387 | ICP 13138 | - | Landrace | Kenya | Africa | https://doi.org/10.18730/Q7A8~ |
| 388 | ICP 13173 | - | Landrace | Kenya | Africa | https://doi.org/10.18730/Q7BBZ |
| 389 | ICP 13194 | Quantum; QPL 42 | Improved cultivar | Australia | Oceania | https://doi.org/10.18730/Q7C0F |
| 390 | ICP 13249 | - | Landrace | Kenya | Africa | https://doi.org/10.18730/Q7DQ~ |
| 391 | ICP 13250 | - | Landrace | Kenya | Africa | https://doi.org/10.18730/Q7DR$ |
| 392 | ICP 13259 | - | Landrace | Kenya | Africa | https://doi.org/10.18730/Q7E16 |
| 393 | ICP 13264 | - | Landrace | Kenya | Africa | https://doi.org/10.18730/Q7E6B |
| 394 | ICP 13267 | - | Landrace | Kenya | Africa | https://doi.org/10.18730/Q7E9E |
| 395 | ICP 13305 | WS 80-287 | Landrace | Italy | Europe | https://doi.org/10.18730/Q7FFF |
| 396 | ICP 13315 | - | Landrace | Rwanda | Africa | https://doi.org/10.18730/Q7FSS |
| 397 | ICP 13316 | - | Landrace | Rwanda | Africa | https://doi.org/10.18730/Q7FTT |
| 398 | ICP 13317 | - | Landrace | Rwanda | Africa | https://doi.org/10.18730/Q7FVV |
| 399 | ICP 13318 | - | Landrace | Rwanda | Africa | https://doi.org/10.18730/Q7FWW |
| 400 | ICP 13357 | - | Landrace | Malawi | Africa | https://doi.org/10.18730/Q7H3Y |
| 401 | ICP 13410 | - | Landrace | Malawi | Africa | https://doi.org/10.18730/Q7JR9 |
| 402 | ICP 13415 | - | Landrace | Malawi | Africa | https://doi.org/10.18730/Q7JXE |
| 403 | ICP 13480 | - | Landrace | Malawi | Africa | https://doi.org/10.18730/Q7MY5 |
| 404 | ICP 13486 | - | Landrace | Malawi | Africa | https://doi.org/10.18730/Q7N4B |
| 405 | ICP 13542 | CPC 13 | Landrace | Montserrat | America | https://doi.org/10.18730/Q7PWY |
| 406 | ICP 13543 | CPC 15 | Landrace | Montserrat | America | https://doi.org/10.18730/Q7PXZ |
| 407 | ICP 13544 | CPC 16 | Landrace | Montserrat | America | https://doi.org/10.18730/Q7PY* |
| 408 | ICP 13545 | CPC 17 | Landrace | Antigua and Barbuda | America | https://doi.org/10.18730/Q7PZ~ |
| 409 | ICP 13546 | CPC 22 | Landrace | Trinidad and Tobago | America | https://doi.org/10.18730/Q7Q0$ |
| 410 | ICP 13547 | CPC 28 | Landrace | Saint Vincent and the Grenadines | America | https://doi.org/10.18730/Q7Q1= |
| 411 | ICP 13550 | CPC 49 | Landrace | Montserrat | America | https://doi.org/10.18730/Q7Q41 |
| 412 | ICP 13551 | CPC 50 | Landrace | Antigua and Barbuda | America | https://doi.org/10.18730/Q7Q52 |
| 413 | ICP 13553 | CPC 62 | Landrace | Saint Kitts and Nevis | America | https://doi.org/10.18730/Q7Q74 |
| 414 | ICP 13557 | CPC 74 | Landrace | Saint Kitts and Nevis | America | https://doi.org/10.18730/Q7QB8 |
| 415 | ICP 13562 | - | Landrace | Ethiopia | Africa | https://doi.org/10.18730/Q7QGD |
| 416 | ICP 13574 | - | Landrace | Sierra Leone | Africa | https://doi.org/10.18730/Q7QWS |
| 417 | ICP 13575 | - | Landrace | Sierra Leone | Africa | https://doi.org/10.18730/Q7QXT |
| 418 | ICP 13576 | - | Landrace | Sierra Leone | Africa | https://doi.org/10.18730/Q7QYV |
| 419 | ICP 13583 | - | Landrace | Malawi | Africa | https://doi.org/10.18730/Q7R5$ |
| 420 | ICP 13591 | - | Landrace | Kenya | Africa | https://doi.org/10.18730/Q7RD5 |
| 421 | ICP 13622 | - | Landrace | Ethiopia | Africa | https://doi.org/10.18730/Q7SCU |
| 422 | ICP 13623 | - | Landrace | Ethiopia | Africa | https://doi.org/10.18730/Q7SD0 |
| 423 | ICP 13624 | - | Landrace | Ethiopia | Africa | https://doi.org/10.18730/Q7SE1 |
| 424 | ICP 13628 | - | Landrace | Ethiopia | Africa | https://doi.org/10.18730/Q7SJ5 |
| 425 | ICP 13637 | - | Landrace | Zambia | Africa | https://doi.org/10.18730/Q7SVE |
| 426 | ICP 13645 | - | Landrace | South Africa | Africa | https://doi.org/10.18730/Q7T3P |
| 427 | ICP 13704 | - | Landrace | Puerto Rico | America | https://doi.org/10.18730/Q7VY7 |
| 428 | ICP 13707 | - | Landrace | Puerto Rico | America | https://doi.org/10.18730/Q7W1A |
| 429 | ICP 13714 | - | Landrace | Puerto Rico | America | https://doi.org/10.18730/Q7W8H |
| 430 | ICP 13749 | - | Landrace | Trinidad and Tobago | America | https://doi.org/10.18730/Q7XBF |
| 431 | ICP 13752 | - | Landrace | Trinidad and Tobago | America | https://doi.org/10.18730/Q7XEJ |
| 432 | ICP 13757 | - | Landrace | Trinidad and Tobago | America | https://doi.org/10.18730/Q7XKQ |
| 433 | ICP 13758 | - | Landrace | Trinidad and Tobago | America | https://doi.org/10.18730/Q7XMR |
| 434 | ICP 13759 | - | Landrace | Trinidad and Tobago | America | https://doi.org/10.18730/Q7XNS |
| 435 | ICP 13760 | - | Landrace | Trinidad and Tobago | America | https://doi.org/10.18730/Q7XPT |
| 436 | ICP 13781 | - | Landrace | Trinidad and Tobago | America | https://doi.org/10.18730/Q7YBA |
| 437 | ICP 13802 | - | Landrace | Trinidad and Tobago | America | https://doi.org/10.18730/Q7Z0Z |
| 438 | ICP 13807 | - | Landrace | Trinidad and Tobago | America | https://doi.org/10.18730/Q7Z5U |
| 439 | ICP 13818 | - | Landrace | Trinidad and Tobago | America | https://doi.org/10.18730/Q7ZGA |
| 440 | ICP 13820 | - | Landrace | Grenada | America | https://doi.org/10.18730/Q7ZJC |
| 441 | ICP 13828 | - | Landrace | Grenada | America | https://doi.org/10.18730/Q7ZTM |
| 442 | ICP 13844 | - | Landrace | Barbados | America | https://doi.org/10.18730/Q80AU |
| 443 | ICP 13846 | - | Landrace | Barbados | America | https://doi.org/10.18730/Q80C1 |
| 444 | ICP 13847 | - | Landrace | Barbados | America | https://doi.org/10.18730/Q80D2 |
| 445 | ICP 13849 | - | Landrace | Barbados | America | https://doi.org/10.18730/Q80F4 |
| 446 | ICP 13855 | - | Landrace | Saint Vincent and the Grenadines | America | https://doi.org/10.18730/Q80NA |
| 447 | ICP 13857 | - | Landrace | Saint Vincent and the Grenadines | America | https://doi.org/10.18730/Q80QC |
| 448 | ICP 13859 | - | Landrace | Saint Vincent and the Grenadines | America | https://doi.org/10.18730/Q80SE |
| 449 | ICP 13860 | - | Landrace | Saint Vincent and the Grenadines | America | https://doi.org/10.18730/Q80TF |
| 450 | ICP 13874 | - | Landrace | Saint Lucia | America | https://doi.org/10.18730/Q818X |
| 451 | ICP 13875 | - | Landrace | Saint Lucia | America | https://doi.org/10.18730/Q819Y |
| 452 | ICP 13876 | - | Landrace | Saint Lucia | America | https://doi.org/10.18730/Q81AZ |
| 453 | ICP 13878 | - | Landrace | Puerto Rico | America | https://doi.org/10.18730/Q81C~ |
| 454 | ICP 13879 | - | Landrace | Puerto Rico | America | https://doi.org/10.18730/Q81D$ |
| 455 | ICP 13880 | - | Landrace | Puerto Rico | America | https://doi.org/10.18730/Q81E= |
| 456 | ICP 13881 | - | Landrace | Puerto Rico | America | https://doi.org/10.18730/Q81FU |
| 457 | ICP 13882 | - | Landrace | Puerto Rico | America | https://doi.org/10.18730/Q81G0 |
| 458 | ICP 13883 | - | Landrace | Puerto Rico | America | https://doi.org/10.18730/Q81H1 |
| 459 | ICP 13884 | - | Landrace | Puerto Rico | America | https://doi.org/10.18730/Q81J2 |
| 460 | ICP 13885 | - | Landrace | Puerto Rico | America | https://doi.org/10.18730/Q81K3 |
| 461 | ICP 13889 | - | Landrace | Dominican Republic | America | https://doi.org/10.18730/Q81Q7 |
| 462 | ICP 13911 | PDGA 67 | Landrace | India | Asia | https://doi.org/10.18730/Q82DX |
| 463 | ICP 13964 | - | Landrace | Dominican Republic | America | https://doi.org/10.18730/Q8428 |
| 464 | ICP 13982 | - | Landrace | Saint Lucia | America | https://doi.org/10.18730/Q84MT |
| 465 | ICP 13988 | - | Landrace | Saint Lucia | America | https://doi.org/10.18730/Q84T* |
| 466 | ICP 13994 | - | Landrace | Bangladesh | Asia | https://doi.org/10.18730/Q8501 |
| 467 | ICP 13998 | - | Landrace | South Africa | Africa | https://doi.org/10.18730/Q8545 |
| 468 | ICP 13999 | - | Landrace | Rwanda | Africa | https://doi.org/10.18730/Q8556 |
| 469 | ICP 14038 | - | Landrace | India | Asia | https://doi.org/10.18730/Q86C8 |
| 470 | ICP 14057 | Hunt, Megha | Improved cultivar | Australia | Oceania | https://doi.org/10.18730/Q86ZV |
| 471 | ICP 14059 | - | Landrace | Guyana | America | https://doi.org/10.18730/Q871X |
| 472 | ICP 14071 | - | Landrace | Guyana | America | https://doi.org/10.18730/Q87D4 |
| 473 | ICP 14099 | - | Landrace | Venezuela | America | https://doi.org/10.18730/Q889* |
| 474 | ICP 14109 | - | Landrace | Jamaica | America | https://doi.org/10.18730/Q88K5 |
| 475 | ICP 14110 | - | Landrace | Jamaica | America | https://doi.org/10.18730/Q88M6 |
| 476 | ICP 14111 | - | Landrace | Jamaica | America | https://doi.org/10.18730/Q88N7 |
| 477 | ICP 14114 | - | Landrace | Jamaica | America | https://doi.org/10.18730/Q88RA |
| 478 | ICP 14165 | - | Landrace | Saint Lucia | America | https://doi.org/10.18730/Q8ABR |
| 479 | ICP 14166 | LRG 36 (Lam redgram) | Improved cultivar | India | Asia | https://doi.org/10.18730/Q8ACS |
| 480 | ICP 14167 | - | Landrace | Venezuela | America | https://doi.org/10.18730/Q8ADT |
| 481 | ICP 14169 | - | Landrace | Jamaica | America | https://doi.org/10.18730/Q8AFW |
| 482 | ICP 14175 | EEI 85032 | Landrace | Brazil | America | https://doi.org/10.18730/Q8AN$ |
| 483 | ICP 14203 | - | Landrace | India | Asia | https://doi.org/10.18730/Q8BHS |
| 484 | ICP 14205 | - | Landrace | India | Asia | https://doi.org/10.18730/Q8BKV |
| 485 | ICP 14230 | ZCC 4 | Landrace | Zambia | Africa | https://doi.org/10.18730/Q8CCF |
| 486 | ICP 14232 | ZCC 6 | Landrace | Zambia | Africa | https://doi.org/10.18730/Q8CEH |
| 487 | ICP 14233 | ZCC 7 | Landrace | Zambia | Africa | https://doi.org/10.18730/Q8CFJ |
| 488 | ICP 14234 | ZCC 8 | Landrace | Zambia | Africa | https://doi.org/10.18730/Q8CGK |
| 489 | ICP 14235 | ZCC 9 | Landrace | Zambia | Africa | https://doi.org/10.18730/Q8CHM |
| 490 | ICP 14242 | ZCC 17 | Landrace | Zambia | Africa | https://doi.org/10.18730/Q8CRV |
| 491 | ICP 14244 | ZCC 19 | Landrace | Zambia | Africa | https://doi.org/10.18730/Q8CTX |
| 492 | ICP 14247 | ZCC 22 | Landrace | Zambia | Africa | https://doi.org/10.18730/Q8CX* |
| 493 | ICP 14249 | ZCC 25 | Landrace | Zambia | Africa | https://doi.org/10.18730/Q8CZ$ |
| 494 | ICP 14254 | ZCC 30 | Landrace | Zambia | Africa | https://doi.org/10.18730/Q8D42 |
| 495 | ICP 14259 | ZCC 37 | Landrace | Zambia | Africa | https://doi.org/10.18730/Q8D97 |
| 496 | ICP 14260 | ZCC 40 | Landrace | Zambia | Africa | https://doi.org/10.18730/Q8DA8 |
| 497 | ICP 14263 | ZCC 54 | Landrace | Zambia | Africa | https://doi.org/10.18730/Q8DDB |
| 498 | ICP 14264 | ZCC 55 | Landrace | Zambia | Africa | https://doi.org/10.18730/Q8DEC |
| 499 | ICP 14266 | ZCC 60 | Landrace | Zambia | Africa | https://doi.org/10.18730/Q8DGE |
| 500 | ICP 14296 | PCC 2 (84) | Landrace | Italy | Europe | https://doi.org/10.18730/Q8EE7 |
| 501 | ICP 14297 | PCC 3 (84) | Landrace | Italy | Europe | https://doi.org/10.18730/Q8EF8 |
| 502 | ICP 14298 | PCC 5 (84) | Landrace | Italy | Europe | https://doi.org/10.18730/Q8EG9 |
| 503 | ICP 14299 | PCC 6 (84) | Landrace | Italy | Europe | https://doi.org/10.18730/Q8EHA |
| 504 | ICP 14334 | ANT 183 | Landrace | Venezuela | America | https://doi.org/10.18730/Q8FM8 |
| 505 | ICP 14337 | MER 194 | Landrace | Venezuela | America | https://doi.org/10.18730/Q8FQB |
| 506 | ICP 14340 | ANT 200 | Landrace | Venezuela | America | https://doi.org/10.18730/Q8FTE |
| 507 | ICP 14360 | BAR 366 | Landrace | Venezuela | America | https://doi.org/10.18730/Q8GE$ |
| 508 | ICP 14378 | PRS 6616 | Landrace | Venezuela | America | https://doi.org/10.18730/Q8H0F |
| 509 | ICP 14379 | PRS 6617 | Landrace | Venezuela | America | https://doi.org/10.18730/Q8H1G |
| 510 | ICP 14380 | PRS 6621 | Landrace | Venezuela | America | https://doi.org/10.18730/Q8H2H |
| 511 | ICP 14387 | Guamo | Landrace | Venezuela | America | https://doi.org/10.18730/Q8H9R |
| 512 | ICP 14388 | - | Landrace | Central African Republic | Africa | https://doi.org/10.18730/Q8HAS |
| 513 | ICP 14389 | - | Landrace | Central African Republic | Africa | https://doi.org/10.18730/Q8HBT |
| 514 | ICP 14390 | KLM 1870 | Landrace | Maldives | Asia | https://doi.org/10.18730/Q8HCV |
| 515 | ICP 14497 | - | Landrace | India | Asia | https://doi.org/10.18730/Q8MQQ |
| 516 | ICP 14515 | - | Landrace | India | Asia | https://doi.org/10.18730/Q8N94 |
| 517 | ICP 14521 | - | Landrace | India | Asia | https://doi.org/10.18730/Q8NFA |
| 518 | ICP 14530 | - | Landrace | India | Asia | https://doi.org/10.18730/Q8NRK |
| 519 | ICP 14532 | - | Landrace | India | Asia | https://doi.org/10.18730/Q8NTN |
| 520 | ICP 14537 | - | Landrace | India | Asia | https://doi.org/10.18730/Q8NZT |
| 521 | ICP 14557 | No. 0004 | Landrace | Thailand | Asia | https://doi.org/10.18730/Q8PK9 |
| 522 | ICP 14561 | No. 0048 | Landrace | Thailand | Asia | https://doi.org/10.18730/Q8PQD |
| 523 | ICP 14562 | No. 0057 | Landrace | Thailand | Asia | https://doi.org/10.18730/Q8PRE |
| 524 | ICP 14564 | No. 120 | Landrace | Thailand | Asia | https://doi.org/10.18730/Q8PTG |
| 525 | ICP 14565 | No. 127 | Landrace | Thailand | Asia | https://doi.org/10.18730/Q8PVH |
| 526 | ICP 14573 | No. 173 | Landrace | Thailand | Asia | https://doi.org/10.18730/Q8Q3S |
| 527 | ICP 14574 | No. 178 | Landrace | Thailand | Asia | https://doi.org/10.18730/Q8Q4T |
| 528 | ICP 14587 | ZCC 41 | Landrace | Zambia | Africa | https://doi.org/10.18730/Q8QH2 |
| 529 | ICP 14588 | ZCC 42 | Landrace | Zambia | Africa | https://doi.org/10.18730/Q8QJ3 |
| 530 | ICP 14598 | - | Landrace | India | Asia | https://doi.org/10.18730/Q8QWD |
| 531 | ICP 14599 | - | Landrace | India | Asia | https://doi.org/10.18730/Q8QXE |
| 532 | ICP 14600 | - | Landrace | India | Asia | https://doi.org/10.18730/Q8QYF |
| 533 | ICP 14601 | - | Landrace | India | Asia | https://doi.org/10.18730/Q8QZG |
| 534 | ICP 14615 | - | Landrace | India | Asia | https://doi.org/10.18730/Q8RDY |
| 535 | ICP 14618 | - | Landrace | India | Asia | https://doi.org/10.18730/Q8RG~ |
| 536 | ICP 14861 | - | Landrace | Indonesia | Asia | https://doi.org/10.18730/Q903H |
| 537 | ICP 14866 | - | Landrace | Myanmar | Asia | https://doi.org/10.18730/Q908P |
| 538 | ICP 14871 | - | Landrace | India | Asia | https://doi.org/10.18730/Q90DV |
| 539 | ICP 14882 | Kerala collection | Landrace | India | Asia | https://doi.org/10.18730/Q90R1 |
| 540 | ICP 14967 | - | Landrace | India | Asia | https://doi.org/10.18730/Q93DC |
| 541 | ICP 15099 | - | Landrace | Uganda | Africa | https://doi.org/10.18730/Q97H~ |
| 542 | ICP 15106 | - | Landrace | Uganda | Africa | https://doi.org/10.18730/Q97R3 |
| 543 | ICP 15108 | - | Landrace | Uganda | Africa | https://doi.org/10.18730/Q97T5 |
| 544 | ICP 15109 | - | Landrace | Uganda | Africa | https://doi.org/10.18730/Q97V6 |
| 545 | ICP 15120 | - | Landrace | Uganda | Africa | https://doi.org/10.18730/Q986H |
| 546 | ICP 15130 | GIN 89-328 | Landrace | United Kingdom | Europe | https://doi.org/10.18730/Q98GV |
| 547 | ICP 15131 | GIN 89-439 | Landrace | United Kingdom | Europe | https://doi.org/10.18730/Q98HW |
| 548 | ICP 15132 | GIN 89-504 | Landrace | United Kingdom | Europe | https://doi.org/10.18730/Q98JX |
| 549 | ICP 15133 | GIN 89-677 | Landrace | United Kingdom | Europe | https://doi.org/10.18730/Q98KY |
| 550 | ICP 15144 | - | Landrace | Congo | Africa | https://doi.org/10.18730/Q98Y4 |
| 551 | ICP 15147 | - | Landrace | Congo | Africa | https://doi.org/10.18730/Q9917 |
| 552 | ICP 15233 | - | Landrace | India | Asia | https://doi.org/10.18730/Q9BQK |
| 553 | ICP 15235 | - | Landrace | India | Asia | https://doi.org/10.18730/Q9BSN |
| 554 | ICP 15237 | - | Landrace | India | Asia | https://doi.org/10.18730/Q9BVQ |
| 555 | ICP 15239 | - | Landrace | India | Asia | https://doi.org/10.18730/Q9BXS |
| 556 | ICP 15240 | - | Landrace | India | Asia | https://doi.org/10.18730/Q9BYT |
| 557 | ICP 15241 | - | Landrace | India | Asia | https://doi.org/10.18730/Q9BZV |
| 558 | ICP 15242 | - | Landrace | India | Asia | https://doi.org/10.18730/Q9C0W |
| 559 | ICP 15245 | - | Landrace | India | Asia | https://doi.org/10.18730/Q9C3Z |
| 560 | ICP 15247 | - | Landrace | India | Asia | https://doi.org/10.18730/Q9C5~ |
| 561 | ICP 15248 | - | Landrace | India | Asia | https://doi.org/10.18730/Q9C6$ |
| 562 | ICP 15249 | - | Landrace | India | Asia | https://doi.org/10.18730/Q9C7= |
| 563 | ICP 15266 | - | Landrace | India | Asia | https://doi.org/10.18730/Q9CRF |
| 564 | ICP 15269 | - | Landrace | India | Asia | https://doi.org/10.18730/Q9CVJ |
| 565 | ICP 15278 | - | Landrace | India | Asia | https://doi.org/10.18730/Q9D4V |
| 566 | ICP 15282 | - | Landrace | India | Asia | https://doi.org/10.18730/Q9D8Z |
| 567 | ICP 15283 | - | Landrace | India | Asia | https://doi.org/10.18730/Q9D9* |
| 568 | ICP 15331 | Collection 16 | Landrace | Myanmar | Asia | https://doi.org/10.18730/Q9ES6 |
| 569 | ICP 15335 | - | Landrace | Nigeria | Africa | https://doi.org/10.18730/Q9EXA |
| 570 | ICP 15336 | - | Landrace | Nigeria | Africa | https://doi.org/10.18730/Q9EYB |
| 571 | ICP 15342 | - | Landrace | Nigeria | Africa | https://doi.org/10.18730/Q9F4H |
| 572 | ICP 15343 | - | Landrace | Nigeria | Africa | https://doi.org/10.18730/Q9F5J |
| 573 | ICP 15344 | - | Landrace | Uganda | Africa | https://doi.org/10.18730/Q9F6K |
| 574 | ICP 15350 | - | Landrace | Uganda | Africa | https://doi.org/10.18730/Q9FCS |
| 575 | ICP 15362 | - | Landrace | Uganda | Africa | https://doi.org/10.18730/Q9FR0 |
| 576 | ICP 15386 | - | Landrace | Nigeria | Africa | https://doi.org/10.18730/Q9GGR |
| 577 | ICP 15387 | - | Landrace | Nigeria | Africa | https://doi.org/10.18730/Q9GHS |
| 578 | ICP 15388 | - | Landrace | Nigeria | Africa | https://doi.org/10.18730/Q9GJT |
| 579 | ICP 15438 | - | Landrace | Nigeria | Africa | https://doi.org/10.18730/Q9J42 |
| 580 | ICP 15489 | Collection 6 | Landrace | Myanmar | Asia | https://doi.org/10.18730/Q9KQG |
| 581 | ICP 15490 | - | Landrace | Uganda | Africa | https://doi.org/10.18730/Q9KRH |
| 582 | ICP 15496 | - | Landrace | Uganda | Africa | https://doi.org/10.18730/Q9KYQ |
| 583 | ICP 15511 | - | Landrace | Uganda | Africa | https://doi.org/10.18730/Q9MD1 |
| 584 | ICP 15528 | - | Landrace | Uganda | Africa | https://doi.org/10.18730/Q9MYJ |
| 585 | ICP 15532 | - | Landrace | Uganda | Africa | https://doi.org/10.18730/Q9N2P |
| 586 | ICP 15597 | MN 1 | Improved cultivar | India | Asia | https://doi.org/10.18730/Q9Q3D |
| 587 | ICP 15611 | ICPW 010; No. 1155 | Wild | Australia | Oceania | https://doi.org/10.18730/Q9QHV |
| 588 | ICP 15613 | ICPW 012; NT 7521 | Wild | Australia | Oceania | https://doi.org/10.18730/Q9QKX |
| 589 | ICP 15632 | ICPW 031 | Wild | India | Asia | https://doi.org/10.18730/Q9R6B |
| 590 | ICP 15761 | ICPW 160 | Wild | India | Asia | https://doi.org/10.18730/Q9W2R |
| 591 | ICP 16344 | PI 304646 | Landrace | Argentina | America | https://doi.org/10.18730/QACDX |
| 592 | ICP 16345 | PI 311502 | Landrace | Brazil | America | https://doi.org/10.18730/QACEY |
| 593 | ICP 16542 | PI 426760 | Landrace | Pakistan | Asia | https://doi.org/10.18730/QAJK5 |
| 594 | ICP 16543 | PI 426761 | Landrace | Pakistan | Asia | https://doi.org/10.18730/QAJM6 |
| 595 | ICP 16677 | PI 398176 | Landrace | India | Asia | https://doi.org/10.18730/QAPTX |
| 596 | ICP 16830 | TZ accession 5; EC 616337 | Landrace | Tanzania | Africa | https://doi.org/10.18730/QAVK$ |
| 597 | ICP 16831 | TZ accession 6; EC 616338 | Landrace | Tanzania | Africa | https://doi.org/10.18730/QAVM= |
| 598 | ICP 16840 | TZ accession 40; EC 616372 | Landrace | Tanzania | Africa | https://doi.org/10.18730/QAVX7 |
| 599 | ICP 16844 | TZ accession 50; EC 616382 | Landrace | Tanzania | Africa | https://doi.org/10.18730/QAW1B |
| 600 | ICP 16848 | TZ accession 62; EC 616394 | Landrace | Tanzania | Africa | https://doi.org/10.18730/QAW5F |
| **Checks** | | | | | | |
| 601 | ICP 8863 | Maruti | Improved cultivar | India | Asia | https://doi.org/10.18730/Q34ND |
| 602 | ICP 7221 | Gwalior 3 | Improved cultivar | India | Asia | https://doi.org/10.18730/Q1HK7 |
| 603 | ICP 6971 | UPAS 120 | Improved cultivar | India | Asia | https://doi.org/10.18730/Q19SG |
| 604 | ICP 11543 | Pragati; ICPL 87 | Improved cultivar | India | Asia | https://doi.org/10.18730/Q5RDX |

**Supplementary Table 2.** Correlation among agronomic traits and grain nutrients of 598 pigeonpea accessions evaluated during the 2019 rainy and 2020 rainy seasons at ICRISAT, India.

|  | **DM** | **SW** | **GYP** | **Protein** | **P** | **K** | **Ca** | **Mg** | **Cu** | **Mn** | **Fe** | **Zn** |
| --- | --- | --- | --- | --- | --- | --- | --- | --- | --- | --- | --- | --- |
| **DFF** | 0.988^**^ | 0.192^**^ | 0.146^**^ | -0.084^*^ | -0.042 | -0.049 | -0.007 | -0.142^**^ | -0.146^**^ | 0.078 | -0.307^**^ | -0.305^**^ |
| **DM** |  | 0.200^**^ | 0.143^**^ | -0.094^*^ | -0.040 | -0.049 | 0.000 | -0.143^**^ | -0.141^**^ | 0.083^*^ | -0.305^**^ | -0.312^**^ |
| **SW** |  |  | -0.254^**^ | -0.370^**^ | 0.076 | 0.158^**^ | -0.180^**^ | -0.253 | 0.403^**^ | -0.140 | -0.175^**^ | -0.185^**^ |
| **GYP** |  |  |  | 0.104^*^ | -0.248^**^ | -0.232^**^ | -0.002 | 0.107^**^ | -0.377^**^ | 0.046 | -0.106^**^ | -0.236^**^ |
| **Protein** |  |  |  |  | 0.306^**^ | 0.242^**^ | -0.018 | 0.300^**^ | -0.024 | 0.136^**^ | 0.327^**^ | 0.429^**^ |
| **P** |  |  |  |  |  | 0.221^**^ | 0.051 | 0.156^**^ | 0.409^**^ | 0.075 | 0.205^**^ | 0.402^**^ |
| **K** |  |  |  |  |  |  | -0.235^**^ | 0.011 | 0.438^**^ | -0.030 | 0.283^**^ | 0.384^**^ |
| **Ca** |  |  |  |  |  |  |  | 0.455^**^ | -0.007 | 0.683^**^ | 0.115^**^ | 0.060 |
| **Mg** |  |  |  |  |  |  |  |  | 0.103^*^ | 0.475^**^ | 0.251^**^ | 0.247^**^ |
| **Cu** |  |  |  |  |  |  |  |  |  | 0.060 | 0.340^**^ | 0.495^**^ |
| **Mn** |  |  |  |  |  |  |  |  |  |  | 0.211^**^ | 0.148^**^ |
| **Fe** |  |  |  |  |  |  |  |  |  |  |  | 0.580^**^ |

DFF, Days to 50% flowering; DM, Days to maturity; SW, 100-seed weight; GYP, Grain yield per plant; P, Phosphorus; K, Potassium; Ca, Calcium; Cu, Copper; Mg, Magnesium; Mn, Manganese; Fe, Iron; Zn, Zinc. ^*^Significant correlation at P≤0.05 and ^**^significant correlation at P≤0.01

**Supplementary Table 3.** Correlation between agronomic traits and grain nutrients for accessions from Asian region

|  | **DM** | **SW** | **GYP** | **Protein** | **P** | **K** | **Ca** | **Mg** | **Cu** | **Mn** | **Fe** | **Zn** |
| --- | --- | --- | --- | --- | --- | --- | --- | --- | --- | --- | --- | --- |
| **DFF** | 0.990^**^ | 0.031 | 0.333^**^ | -0.04 | -0.189^**^ | -0.172^**^ | -0.028 | -0.115^*^ | -0.306^**^ | 0.09 | -0.352^**^ | -0.297^**^ |
| **DM** |  | 0.037 | 0.332^**^ | -0.053 | -0.195^**^ | -0.177^**^ | -0.022 | -0.116^*^ | -0.309^**^ | 0.094 | -0.352^**^ | -0.309^**^ |
| **SW** |  |  | 0.037 | -0.292^**^ | -0.092 | -0.036 | -0.173^**^ | -0.067 | 0.222^**^ | -0.146^**^ | -0.170^**^ | -0.171^**^ |
| **GYP** |  |  |  | -0.018 | -0.316^**^ | -0.186^**^ | -0.038 | -0.016 | -0.288^**^ | 0.039 | -0.132^*^ | -0.328^**^ |
| **Protein** |  |  |  |  | 0.359^**^ | 0.302^**^ | -0.089 | 0.247^**^ | 0.085 | 0.125^*^ | 0.361^**^ | 0.413^**^ |
| **P** |  |  |  |  |  | 0.207^**^ | 0.044 | 0.177^**^ | 0.402^**^ | 0.052 | 0.201^**^ | 0.468^**^ |
| **K** |  |  |  |  |  |  | -0.246^**^ | 0.078 | 0.418^**^ | -0.016 | 0.319^**^ | 0.440^**^ |
| **Ca** |  |  |  |  |  |  |  | 0.418^**^ | 0.080 | 0.663^**^ | 0.103 | 0.027 |
| **Mg** |  |  |  |  |  |  |  |  | 0.300^**^ | 0.495^**^ | 0.285^**^ | 0.222^**^ |
| **Cu** |  |  |  |  |  |  |  |  |  | 0.163^**^ | 0.415^**^ | 0.584^**^ |
| **Mn** |  |  |  |  |  |  |  |  |  |  | 0.245^**^ | 0.161^**^ |
| **Fe** |  |  |  |  |  |  |  |  |  |  |  | 0.606^**^ |

DFF, Days to 50% flowering; DM, Days to maturity; SW, 100-seed weight; GYP, Grain yield per plant; P, Phosphorus; K, Potassium; Ca, Calcium; Cu, Copper; Mg, Magnesium; Mn, Manganese; Fe, Iron; Zn, Zinc. ^*^ Significant correlation at P≤0.05 and ^**^ significant correlation at P≤0.01

**Supplementary Table 4.** Correlation between agronomic traits and grain nutrients for accessions from African region

|  | **DM** | **SW** | **GYP** | **Protein** | **P** | **K** | **Ca** | **Mg** | **Cu** | **Mn** | **Fe** | **Zn** |
| --- | --- | --- | --- | --- | --- | --- | --- | --- | --- | --- | --- | --- |
| **DFF** | 0.970^**^ | 0.458^**^ | -0.145 | -0.142 | 0.047 | 0.158 | 0.075 | -0.154 | 0.062 | 0.032 | -0.282^**^ | -0.328^**^ |
| **DM** |  | 0.469^**^ | -0.157 | -0.129 | 0.066 | 0.178^*^ | 0.107 | -0.145 | 0.093 | 0.051 | -0.283^**^ | -0.329^**^ |
| **SW** |  |  | -0.325^**^ | -0.085 | 0.22 | 0.328^**^ | -0.046 | -0.222^**^ | 0.36o^**^ | 0.028 | -0.213^**^ | -0.048 |
| **GYP** |  |  |  | -0.057 | -0.089 | -0.366^**^ | -0.022 | 0.08 | -0.258^**^ | -0.025 | -0.023 | -0.259^**^ |
| **Protein** |  |  |  |  | 0.379^**^ | 0.272^**^ | -0.067 | 0.249^**^ | 0.161^*^ | 0.113 | 0.342^**^ | 0.453^**^ |
| **P** |  |  |  |  |  | 0.099 | 0.096 | 0.219^**^ | 0.368^**^ | 0.115 | 0.158 | 0.301^**^ |
| **K** |  |  |  |  |  |  | -0.245^**^ | -0.045 | 0.417^**^ | 0.019 | 0.206^*^ | 0.337^**^ |
| **Ca** |  |  |  |  |  |  |  | 0.545^**^ | 0.027 | 0.626^**^ | 0.099 | 0.083 |
| **Mg** |  |  |  |  |  |  |  |  | 0.065 | 0.466^**^ | 0.229^**^ | 0.242^**^ |
| **Cu** |  |  |  |  |  |  |  |  |  | 0.112 | 0.188^*^ | 0.532^**^ |
| **Mn** |  |  |  |  |  |  |  |  |  |  | 0.146 | 0.174^*^ |
| **Fe** |  |  |  |  |  |  |  |  |  |  |  | 0.542^**^ |

DFF, Days to 50% flowering; DM, Days to maturity; SW, 100-seed weight; GYP, Grain yield per plant; P, Phosphorus; K, Potassium; Ca, Calcium; Cu, Copper; Mg, Magnesium; Mn, Manganese; Fe, Iron; Zn, Zinc. ^*^ Significant correlation at P≤0.05 and ^**^ significant correlation at P≤0.01

**Supplementary Table 5:** Correlation between agronomic traits and grain nutrients for accessions from American region

|  | **DM** | **SW** | **GYP** | **Protein** | **P** | **K** | **Ca** | **Mg** | **Cu** | **Mn** | **Fe** | **Zn** |
| --- | --- | --- | --- | --- | --- | --- | --- | --- | --- | --- | --- | --- |
| **DFF** | 0.985^**^ | -0.051 | -0.014 | -0.008 | 0.028 | -0.061 | -0.054 | -0.116 | -0.303^**^ | 0.031 | -0.288^**^ | -0.245^*^ |
| **DM** |  | -0.053 | -0.014 | -0.026 | 0.033 | -0.07 | -0.077 | -0.136 | -0.305^**^ | 0.01 | -0.271^*^ | -0.238^*^ |
| **SW** |  |  | -0.214 | -0.304^**^ | 0.003 | 0.29 | -0.285^*^ | -0.359^**^ | 0.184 | -0.245^*^ | -0.365^**^ | -0.19 |
| **GYP** |  |  |  | 0.125 | -0.155 | -0.118 | -0.115 | 0.103 | -0.346^**^ | -0.098 | -0.189 | -0.241^*^ |
| **Protein** |  |  |  |  | 0.387^**^ | 0.323^**^ | 0.117 | 0.255^*^ | 0.176 | 0.046 | 0.219^*^ | 0.424^**^ |
| **P** |  |  |  |  |  | 0.447^**^ | -0.004 | 0.168 | 0.592^**^ | 0.035 | 0.274^*^ | 0.573^**^ |
| **K** |  |  |  |  |  |  | -0.239^*^ | -0.006 | 0.545^**^ | -0.187 | 0.226^*^ | 0.370^**^ |
| **Ca** |  |  |  |  |  |  |  | 0.399^**^ | -0.09 | 0.812^**^ | 0.184 | 0.109 |
| **Mg** |  |  |  |  |  |  |  |  | 0.16 | 0.332^**^ | 0.245^*^ | 0.240^*^ |
| **Cu** |  |  |  |  |  |  |  |  |  | -0.094 | 0.488^**^ | 0.682^**^ |
| **Mn** |  |  |  |  |  |  |  |  |  |  | 0.132 | 0.036 |
| **Fe** |  |  |  |  |  |  |  |  |  |  |  | 0.608^**^ |

DFF, Days to 50% flowering; DM, Days to maturity; SW, 100-seed weight; GYP, Grain yield per plant; P, Phosphorus; K, Potassium; Ca, Calcium; Cu, Copper; Mg, Magnesium; Mn, Manganese; Fe, Iron; Zn, Zinc. ^*^ Significant correlation at P≤0.05 and ^**^ significant correlation at P≤0.01

**Supplementary Table 6:** Correlation between agronomic traits and grain nutrients for accessions from early maturity group

|  | **DM** | **SW** | **GYP** | **Protein** | **P** | **K** | **Ca** | **Mg** | **Cu** | **Mn** | **Fe** | **Zn** |
| --- | --- | --- | --- | --- | --- | --- | --- | --- | --- | --- | --- | --- |
| **DFF** | 0.943^**^ | 0.295 | 0.459^**^ | 0.239 | -0.163 | 0.163 | -0.103 | 0.141 | -0.185 | 0.123 | 0.236 | -0.111 |
| **DM** |  | 0.365^*^ | 0.430^*^ | 0.161 | -0.167 | 0.167 | -0.131 | 0.12 | -0.187 | 0.026 | 0.19 | -0.135 |
| **SW** |  |  | 0.092 | 0.138 | -0.068 | -0.021 | -0.452^**^ | -0.226 | -0.294 | -0.319 | -0.144 | -0.236 |
| **GYP** |  |  |  | 0.392^*^ | -0.256 | -0.117 | -0.221 | -0.016 | -0.592^**^ | -0.224 | -0.055 | -0.493^**^ |
| **Protein** |  |  |  |  | 0.280 | 0.253 | -0.024 | 0.325 | -0.176 | 0.107 | 0.146 | -0.006 |
| **P** |  |  |  |  |  | 0.246 | 0.215 | 0.516^**^ | 0.400^*^ | 0.361^*^ | -0.014 | 0.365^*^ |
| **K** |  |  |  |  |  |  | 0.04 | 0.424^*^ | 0.425^*^ | 0.279 | 0.550^**^ | 0.409^*^ |
| **Ca** |  |  |  |  |  |  |  | 0.424^*^ | 0.148 | 0.794^**^ | 0.124 | 0.222 |
| **Mg** |  |  |  |  |  |  |  |  | 0.428^*^ | 0.537^**^ | 0.151 | 0.29 |
| **Cu** |  |  |  |  |  |  |  |  |  | 0.207 | 0.241 | 0.718^**^ |
| **Mn** |  |  |  |  |  |  |  |  |  |  | 0.287 | 0.228 |
| **Fe** |  |  |  |  |  |  |  |  |  |  |  | 0.500^**^ |

DFF, Days to 50% flowering; DM, Days to maturity; SW, 100-seed weight; GYP, Grain yield per plant; P, Phosphorus; K, Potassium; Ca, Calcium; Cu, Copper; Mg, Magnesium; Mn, Manganese; Fe, Iron; Zn, Zinc. ^*^ Significant correlation at P≤0.05 and ^**^ significant correlation at P≤0.01

**Supplementary Table 7.** Correlation between agronomic traits and grain nutrients for accessions from medium duration group

|  | **DM** | **SW** | **GYP** | **Protein** | **P** | **K** | **Ca** | **Mg** | **Cu** | **Mn** | **Fe** | **Zn** |
| --- | --- | --- | --- | --- | --- | --- | --- | --- | --- | --- | --- | --- |
| **DFF** | 0.957^**^ | 0.297^**^ | 0.028 | -0.221^**^ | 0.005 | -0.027 | -0.055 | -0.074 | 0.140^**^ | -0.017 | -0.230^**^ | -0.195^**^ |
| **DM** |  | 0.300^**^ | 0.024 | -0.221^**^ | 0.02 | -0.021 | -0.039 | -0.066 | 0.166^**^ | -0.009 | -0.203^**^ | -0.190^**^ |
| **SW** |  |  | -0.202^**^ | -0.439^**^ | -0.003 | 0.075 | -0.165^**^ | -0.2 | 0.459^**^ | -0.155^**^ | -0.188^**^ | -0.144^**^ |
| **GYP** |  |  |  | 0.097 | -0.203^**^ | -0.252^**^ | 0.049 | 0.049 | -0.340^**^ | 0.04 | -0.142^**^ | -0.249^**^ |
| **Protein** |  |  |  |  | 0.344^**^ | 0.235^**^ | -0.049 | 0.227^**^ | -0.09 | 0.104 | 0.327^**^ | 0.387^**^ |
| **P** |  |  |  |  |  | 0.176^**^ | 0.016 | 0.129 | 0.354^**^ | 0.086 | 0.162^**^ | 0.378^**^ |
| **K** |  |  |  |  |  |  | -0.246^**^ | 0.006 | 0.399^**^ | -0.05 | 0.285^**^ | 0.405^**^ |
| **Ca** |  |  |  |  |  |  |  | 0.473^**^ | -0.027 | 0.728^**^ | 0.073 | 0.009 |
| **Mg** |  |  |  |  |  |  |  |  | 0.067 | 0.481^**^ | 0.207^**^ | 0.146^**^ |
| **Cu** |  |  |  |  |  |  |  |  |  | 0.045 | 0.258^**^ | 0.451^**^ |
| **Mn** |  |  |  |  |  |  |  |  |  |  | 0.229^**^ | 0.125^*^ |
| **Fe** |  |  |  |  |  |  |  |  |  |  |  | 0.567^**^ |

DFF, Days to 50% flowering; DM, Days to maturity; SW, 100-seed weight; GYP, Grain yield per plant; P, Phosphorus; K, Potassium; Ca, Calcium; Cu, Copper; Mg, Magnesium; Mn, Manganese; Fe, Iron; Zn, Zinc. ^*^ Significant correlation at P≤0.05 and ^**^ significant correlation at P≤0.01

**Supplementary Table 8.** Correlation between agronomic traits and grain nutrients for accessions from late maturity group

|  | **DM** | **SW** | **GYP** | **Protein** | **P** | **K** | **Ca** | **Mg** | **Cu** | **Mn** | **Fe** | **Zn** |
| --- | --- | --- | --- | --- | --- | --- | --- | --- | --- | --- | --- | --- |
| **DFF** | 0.920^**^ | 0.088 | -0.240^**^ | 0.08 | 0.098 | 0.088 | 0.106 | -0.051 | 0.130^*^ | 0.172^**^ | -0.100 | 0.137^*^ |
| **DM** |  | 0.136^*^ | -0.266^**^ | 0.013 | 0.087 | 0.074 | 0.143^*^ | -0.066 | 0.130 | 0.190^**^ | -0.143^*^ | 0.079 |
| **SW** |  |  | -0.437^**^ | -0.288^**^ | 0.211^**^ | 0.320^**^ | -0.203^**^ | -0.303^**^ | 0.473^**^ | -0.141^*^ | -0.111 | -0.182^**^ |
| **GYP** |  |  |  | 0.125^*^ | -0.283^**^ | -0.201^**^ | -0.051 | 0.249^**^ | -0.333^**^ | 0.038 | 0.059 | -0.057 |
| **Protein** |  |  |  |  | 0.252^**^ | 0.248^**^ | 0.031 | 0.393^**^ | 0.061 | 0.193^**^ | 0.353^**^ | 0.574^**^ |
| **P** |  |  |  |  |  | 0.276^**^ | 0.085 | 0.151^*^ | 0.475^**^ | 0.056 | 0.258^**^ | 0.442^**^ |
| **K** |  |  |  |  |  |  | -0.262 | -0.04 | 0.494^**^ | -0.017 | 0.229^**^ | 0.349^**^ |
| **Ca** |  |  |  |  |  |  |  | 0.441^**^ | -0.006 | 0.646^**^ | 0.194^**^ | 0.128^*^ |
| **Mg** |  |  |  |  |  |  |  |  | 0.054 | 0.507^**^ | 0.264^**^ | 0.312^**^ |
| **Cu** |  |  |  |  |  |  |  |  |  | 0.113 | 0.357^**^ | 0.429^**^ |
| **Mn** |  |  |  |  |  |  |  |  |  |  | 0.245^**^ | 0.245^**^ |
| **Fe** |  |  |  |  |  |  |  |  |  |  |  | 0.515^**^ |

DFF, Days to 50% flowering; DM, Days to maturity; SW, 100-seed weight; GYP, Grain yield per plant; P, Phosphorus; K, Potassium; Ca, Calcium; Cu, Copper; Mg, Magnesium; Mn, Manganese; Fe, Iron; Zn, Zinc. ^*^ Significant correlation at P≤0.05 and ^**^ significant correlation at P≤0.01

**Supplementary Table 9.** Correlation between agronomic traits and grain nutrients for accessions with ≤10g 100-seed weight

|  | **DM** | **SW** | **GYP** | **Protein** | **P** | **K** | **Ca** | **Mg** | **Cu** | **Mn** | **Fe** | **Zn** |
| --- | --- | --- | --- | --- | --- | --- | --- | --- | --- | --- | --- | --- |
| **DFF** | 0.991^**^ | -0.018 | 0.343^**^ | -0.008 | -0.150^**^ | -0.142^**^ | 0.032 | -0.087 | -0.339^**^ | 0.139^**^ | -0.34^**^ | -0.309^**^ |
| **DM** |  | -0.016 | 0.341^**^ | -0.027 | -0.151^**^ | -0.150^**^ | 0.037 | -0.087 | -0.337^**^ | 0.140^**^ | -0.336^**^ | -0.318^**^ |
| **SW** |  |  | 0.052 | -0.156^**^ | 0.003 | -0.017 | -0.322^**^ | -0.105 | 0.106^*^ | -0.270^**^ | 0.062 | -0.030 |
| **GYP** |  |  |  | 0.054 | -0.263^**^ | -0.206^**^ | -0.053 | -0.009 | -0.338^**^ | 0.048 | -0.157^**^ | -0.331^**^ |
| **Protein** |  |  |  |  | 0.318^**^ | 0.268^**^ | -0.096 | 0.281^**^ | 0.089 | 0.135^*^ | 0.270^**^ | 0.363^**^ |
| **P** |  |  |  |  |  | 0.239^**^ | 0.048 | 0.194^**^ | 0.433^**^ | 0.083 | 0.162^**^ | 0.452^**^ |
| **K** |  |  |  |  |  |  | -0.194^**^ | 0.136^*^ | 0.436^**^ | -0.007 | 0.316^**^ | 0.436^**^ |
| **Ca** |  |  |  |  |  |  |  | 0.415^**^ | 0.064 | 0.671^**^ | 0.098 | 0.064 |
| **Mg** |  |  |  |  |  |  |  |  | 0.288^**^ | 0.464^**^ | 0.261^**^ | 0.274^**^ |
| **Cu** |  |  |  |  |  |  |  |  |  | 0.146^**^ | 0.478^**^ | 0.652^**^ |
| **Mn** |  |  |  |  |  |  |  |  |  |  | 0.205^**^ | 0.188^**^ |
| **Fe** |  |  |  |  |  |  |  |  |  |  |  | 0.588^**^ |

DFF, Days to 50% flowering; DM, Days to maturity; SW, 100-seed weight; GYP, Grain yield per plant; P, Phosphorus; K, Potassium; Ca, Calcium; Cu, Copper; Mg, Magnesium; Mn, Manganese; Fe, Iron; Zn, Zinc. ^*^ Significant correlation at P≤0.05 and ^**^ significant correlation at P≤0.01

**Supplementary Table 10.** Correlation between agronomic traits and grain nutrients for accessions with 10-15g 100-seed weight

|  | **DM** | **SW** | **GYP** | **Protein** | **P** | **K** | **Ca** | **Mg** | **Cu** | **Mn** | **Fe** | **Zn** |
| --- | --- | --- | --- | --- | --- | --- | --- | --- | --- | --- | --- | --- |
| **DFF** | 0.970^**^ | 0.046 | -0.013 | -0.055 | 0.079 | -0.006 | -0.005 | -0.085 | -0.045 | 0.004 | -0.160^*^ | -0.193^**^ |
| **DM** |  | 0.02 | -0.009 | -0.026 | 0.085 | 0.006 | 0.006 | -0.083 | -0.047 | 0.015 | -0.158^*^ | -0.194^**^ |
| **SW** |  |  | -0.18 | -0.222^**^ | 0.135 | 0.082 | -0.046 | -0.098 | 0.287^**^ | -0.106^*^ | -0.125 | -0.03 |
| **GYP** |  |  |  | -0.052 | -0.240^**^ | -0.140^*^ | -0.089 | 0.053 | -0.289^**^ | -0.036 | -0.147^*^ | -0.256^**^ |
| **Protein** |  |  |  |  | 0.457^**^ | 0.428^**^ | -0.037 | 0.175^*^ | 0.177^*^ | 0.051 | 0.337^**^ | 0.444^**^ |
| **P** |  |  |  |  |  | 0.238^**^ | 0.079 | 0.195^**^ | 0.453^**^ | 0.098 | 0.334^**^ | 0.434^**^ |
| **K** |  |  |  |  |  |  | -0.247^**^ | -0.049 | 0.394^**^ | -0.048 | 0.336^**^ | 0.435^**^ |
| **Ca** |  |  |  |  |  |  |  | 0.468^**^ | 0.031 | 0.659^**^ | 0.079 | 0.017 |
| **Mg** |  |  |  |  |  |  |  |  | 0.146^*^ | 0.490^**^ | 0.143^*^ | 0.133 |
| **Cu** |  |  |  |  |  |  |  |  |  | 0.018 | 0.392^**^ | 0.575^**^ |
| **Mn** |  |  |  |  |  |  |  |  |  |  | 0.181^**^ | 0.044 |
| **Fe** |  |  |  |  |  |  |  |  |  |  |  | 0.517^**^ |

DFF, Days to 50% flowering; DM, Days to maturity; SW, 100-seed weight; GYP, Grain yield per plant; P, Phosphorus; K, Potassium; Ca, Calcium; Cu, Copper; Mg, Magnesium; Mn, Manganese; Fe, Iron; Zn, Zinc. ^*^ Significant correlation at P≤0.05 and ^**^ significant correlation at P≤0.01.

**Supplementary Table 11.** Correlation between agronomic traits and grain nutrients for accessions with >15g 100-seed weight

|  | **DM** | **SW** | **GYP** | **Protein** | **P** | **K** | **Ca** | **Mg** | **Cu** | **Mn** | **Fe** | **Zn** |
| --- | --- | --- | --- | --- | --- | --- | --- | --- | --- | --- | --- | --- |
| **DFF** | 0.993^**^ | -0.243 | -0.410^**^ | 0.295 | 0.161 | 0.152 | -0.021 | -0.259 | -0.199 | -0.009 | -0.038 | -0.184 |
| **DM** |  | -0.275 | -0.411^**^ | 0.319 | 0.182 | 0.158 | 0.022 | -0.244 | -0.186 | 0.040 | -0.015 | -0.16 |
| **SW** |  |  | 0.167 | -0.315 | -0.161 | -0.159 | -0.111 | -0.111 | 0.193 | -0.129 | -0.329^*^ | -0.027 |
| **GYP** |  |  |  | -0.255 | -0.098 | -0.438^**^ | 0.321^*^ | 0.508^**^ | -0.133 | 0.119 | -0.251 | -0.29 |
| **Protein** |  |  |  |  | 0.286 | 0.316^*^ | 0.189 | 0.169 | 0.148 | 0.371 | 0.532^**^ | 0.511^**^ |
| **P** |  |  |  |  |  | -0.053 | 0.059 | 0.011 | 0.169 | 0.001 | 0.225 | 0.293 |
| **K** |  |  |  |  |  |  | -0.326^*^ | -0.116 | 0.304 | 0.02 | 0.385^*^ | 0.369 |
| **Ca** |  |  |  |  |  |  |  | 0.558^**^ | -0.123 | 0.836^**^ | 0.184 | -0.087 |
| **Mg** |  |  |  |  |  |  |  |  | -0.088 | 0.503^**^ | 0.263 | 0.006 |
| **Cu** |  |  |  |  |  |  |  |  |  | -0.035 | 0.301 | 0.593^**^ |
| **Mn** |  |  |  |  |  |  |  |  |  |  | 0.284 | 0.080 |
| **Fe** |  |  |  |  |  |  |  |  |  |  |  | 0.603^**^ |

DFF, Days to 50% flowering; DM, Days to maturity; SW, 100-seed weight; GYP, Grain yield per plant; P, Phosphorus; K, Potassium; Ca, Calcium; Cu, Copper; Mg, Magnesium; Mn, Manganese; Fe, Iron; Zn, Zinc. ^*^ Significant correlation at P≤0.05 and ^**^ significant correlation at P≤0.01

**Supplementary Table 12.** Contribution of accessions from different regions to each sub-cluster

| **Region** | **Number of accessions (Percentage of accessions)** | | | | | | **Total** |
| --- | --- | --- | --- | --- | --- | --- | --- |
|  | **Sub-cluster 1** | **Sub-cluster 2** | **Sub-cluster 3** | **Sub-cluster 4** | **Sub-cluster 5** | **Sub-cluster 6** |  |
| **Asia** | 46  (77.97%) | 49  (94.23%) | 109  (87.9%) | 37  (26.81%) | 34  (36.96%) | 83  (62.41%) | 358 |
|  |  |  |  |  |  |  | 59.87% |
| **Africa** | 5  (8.47%) | 3  (5.77%) | 11  (8.87%) | 80  (57.97%) | 18  19.57% | 31  (23.31%) | 148 |
|  |  |  |  |  |  |  | 24.75% |
| **America** | 4  (6.78%) | 0 | 4  (3.23%) | 21  15.22% | 33  35.87% | 17  (12.78%) | 79 |
|  |  |  |  |  |  |  | 13.21% |
| **Europe** | 2  (3.39%) | 0 | 0 | 0 | 7  (7.61%) | 2  (1.50%) | 11 |
|  |  |  |  |  |  |  | 1.84% |
| **Oceania** | 2  (3.39%) | 0 | 0 | 0 | 0 | 0  0 | 2 |
|  |  |  |  |  |  |  | 0.33% |
| **Total** | 59 | 52 | 124 | 138 | 92 | 133 | 598 |

**Supplementary Table 13.** Within and between cluster distances based on Gower’s phenotypic distance matrix for 598 pigeonpea accessions evaluated during the 2019 and 2020 rainy seasons at ICRISAT, India

|  | **Sub-cluster 1** | **Sub-cluster 2** | **Sub-cluster 3** | **Sub-cluster 4** | **Sub-cluster 5** | **Sub-cluster 6** |
| --- | --- | --- | --- | --- | --- | --- |
| **Sub-cluster 1** | **0.136** | 0.187 | 0.166 | 0.187 | 0.186 | 0.227 |
| **Sub-cluster** 2 |  | **0.099** | 0.143 | 0.152 | 0.158 | 0.154 |
| **Sub-cluster** 3 |  |  | **0.124** | 0.169 | 0.144 | 0.164 |
| **Sub-cluster** 4 |  |  |  | **0.135** | 0.149 | 0.165 |
| **Sub-cluster 5** |  |  |  |  | **0.118** | 0.143 |
| **Sub-cluster 6** |  |  |  |  |  | **0.132** |

Diagonal values (bolded) represents the intra-cluster distance

**Supplementary Table 14.** Mean and range comparison for agronomic traits and grain nutrients between six sub-clusters of 598 pigeonpea accessions evaluated during 2019 rainy and 2020 rainy seasons at ICRISAT, India

| **Traits** | **Mean ± SD** | | | | | | **Range** | | | | | | |
| --- | --- | --- | --- | --- | --- | --- | --- | --- | --- | --- | --- | --- | --- |
|  | **Major cluster I** | | | **Major cluster II** | | | **Major cluster I** | | | **Major cluster II** | | | |
|  | **Sub-**  **cluster 1**  **(59)** | **Sub-cluster 2**  **(52)** | **Sub-**  **cluster 3**  **(124)** | **Sub-cluster 4**  **(138)** | **Sub-**  **cluster 5**  **(92)** | **Sub-**  **cluster 6**  **(133)** | **Sub-**  **cluster 1**  **(59)** | **Sub-cluster 2**  **(52)** | **Sub-cluster 3**  **(124)** | **Sub-cluster 4**  **(138)** | **Sub-**  **cluster 5**  **(92)** | | **Sub-cluster 6**  **(133)** |
| **DFF**  **(days)** | 95±13^d^ | 135±5^a^ | 113±7^c^ | 133±10^a^ | 121±9^b^ | 135±8^a^ | 67-122 | 122-146 | 97-134 | 107-166 | 100-146 | 116-150 | |
| **DM**  **(days)** | 146±13^d^ | 185±5^a^ | 163±7^c^ | 183±9^a^ | 172±8^b^ | 185±7^a^ | 112-171 | 174-195 | 148-184 | 160-213 | 150-197 | 167-203 | |
| **SW**  **(g)** | 8.61±  1.8^c^ | 8.27±  0.9^c^ | 8.61±  1.6^c^ | 11.94±  2.7^a^ | 11.8±  3.1^a^ | 9.77±  2.4^b^ | 1.69-  14.96 | 6.46-  10.72 | 6.39-  16.16 | 6.95-  17.78 | 7.04-  22.17 | 5.63-  18.69 | |
| **GYP**  **(g)** | 24.88±  4.2^d^ | 37.64±  6.3^a^ | 36.63±  5.9^a^ | 27.78±  4.7^c^ | 31.05±  4.8^b^ | 35.19±  6.8^a^ | 16.54-32.58 | 27.75-57.93 | 22.75-53.75 | 17.42-41.03 | 16.66-43.23 | 18.83-53.61 | |
| **Protein**  **(%)** | 27.43±  0.8^b^ | 28.15±  0.6^a^ | 27.45±  0.8^b^ | 26.96±  0.7^c^ | 26.15±  0.8^e^ | 26.54±  0.8^d^ | 24.68-29.16 | 26.72-29.02 | 25.56-  29.5 | 24.83-  28.8 | 24.54-28.41 | 23.35-28.42 | |
| **P**  **(%)** | 0.44±  0.02^b^ | 0.43±  0.01^c^ | 0.43±  0.02^c^ | 0.45±  0.02^a^ | 0.42±  0.01^d^ | 0.42±  0.02^d^ | 0.40-  0.48 | 0.40-  0.46 | 0.37  -0.48 | 0.40  -0.50 | 0.38-  0.45 | 0.36-  0.47 | |
| **K**  **(%)** | 1.52±  0.03^a^ | 1.51±  0.02^b^ | 1.5±  0.02^c^ | 1.52±  0.02^a^ | 1.5±  0.02^c^ | 1.49±  0.02^c^ | 1.44-  1.63 | 1.48-  1.54 | 1.43-  1.56 | 1.48-  1.58 | 1.45-  1.54 | 1.43-  1.55 | |
| **Ca**  **(mg/kg)** | 1541.52±  175.8^ab^ | 1552.5±  128.4^ab^ | 1583.83±  194.3^a^ | 1537.76±  157.6^ab^ | 1559.23±  143.1^ab^ | 1495.21±  183.0^b^ | 1188.95-2006.41 | 1315.57-1913.49 | 1081.02-2099.76 | 1042.36-1923.79 | 1224.86-1863.51 | 1098.43-1965.28 | |
| **Mg**  **(mg/kg)** | 1564.98±  57.1^b^ | 1597.43±65.6^a^ | 1551.66±  72.64^b^ | 1523.13±  80.8^c^ | 1525.99±  53.8^c^ | 1478.71±  73.5^d^ | 1426.27-1699.08 | 1489.77-1789.81 | 1362.34-1750.32 | 1342.68-1865.65 | 1413.75-1667.96 | 1311.01-1717.62 | |
| **Cu**  **(mg/kg)** | 11.77±  0.8^a^ | 10.88±  0.5^c^ | 10.84±  0.6c | 11.85±  0.7^a^ | 11.3±  0.6^b^ | 10.53±  0.7^d^ | 10.5-  13.96 | 9.81-  11.78 | 9.46-  12.62 | 10.05-  14.2 | 10.03-13.17 | 7.72-  12.04 | |
| **Mn**  **(mg/kg)** | 10.42±  0.7^bc^ | 10.73±  0.5^a^ | 10.5±  0.6^b^ | 10.44±  0.6^bc^ | 10.29±  0.5^bc^ | 10.21±  0.7c | 9.35-  14.01 | 10.01-12.34 | 9.16-  12.08 | 8.56-  12.45 | 9.22-  11.65 | 9.00-  12.51 | |
| **Fe**  **(mg/kg)** | 36.62±  1.5^a^ | 35.68±  1.4^b^ | 35.08±  1.4^c^ | 35.35±  1.6^bc^ | 34.18±1.25^d^ | 33.67±  1.5^e^ | 33.16-39.81 | 31.74-39.18 | 32.48-39.00 | 31.96-40.98 | 30.00-37.07 | 29.23-37.76 | |
| **Zn**  **(mg/kg)** | 31.21±  1.4^a^ | 30.24±  1.0^b^ | 29.34±  1.2^c^ | 29.71±  1.4^c^ | 28.6±  1.1^d^ | 27.95±  1.1^e^ | 28.23-35.68 | 27.59-  31.5 | 25.6-  32.99 | 26.77-34.18 | 25.51-31.18 | 24.14-31.18 | |

SD, Standard deviation. Values inside the parenthesis () represents the number of accessions in each category. DFF, Days to 50% flowering; DM, Days to maturity; SW, 100-seed weight; SPY, Yield/plant; P, Phosphorus; K, Potassium; Ca, Calcium; Cu, Copper; Mg, Magnesium; Mn, Manganese; Fe, Iron; Zn, Zinc. Mean followed by same letters (across column) are not significant at P ≤ 0.05 and mean followed by different letters (across column) are significant at P ≤ 0.05.
